# Electrospun Ceramic Nanofiber Mats Today: Synthesis, Properties, and Applications

**DOI:** 10.3390/ma10111238

**Published:** 2017-10-27

**Authors:** Hamid Esfahani, Rajan Jose, Seeram Ramakrishna

**Affiliations:** 1 Department of Materials Engineering, Bu-Ali Sina University, Hamedan 65178-38695, Iran; 2 Faculty of Industrial Sciences & Technology, Universiti Malaysia Pahang, Lebuhraya Tun Razak, Gambang 26300, Kuantan, Malaysia; rjose@ump.edu.my; 3 Center for Nanofibers and Nanotechnology, Department of Mechanical Engineering, Faculty of Engineering, 2 Engineering Drive 3, National University of Singapore, Singapore 117576, Singapore; seeram@nus.edu.sg

**Keywords:** electrospinning, nano fabrication, nano ceramic fibers, materials characterization, properties of ceramic materials

## Abstract

Ceramic nanofibers (NFs) have recently been developed for advanced applications due to their unique properties. In this article, we review developments in electrospun ceramic NFs with regard to their fabrication process, properties, and applications. We find that surface activity of electrospun ceramic NFs is improved by post pyrolysis, hydrothermal, and carbothermal processes. Also, when combined with another surface modification methods, electrospun ceramic NFs result in the advancement of properties and widening of the application domains. With the decrease in diameter and length of a fiber, many properties of fibrous materials are modified; characteristics of such ceramic NFs are different from their wide and long (bulk) counterparts. In this article, electrospun ceramic NFs are reviewed with an emphasis on their applications as catalysts, membranes, sensors, biomaterials, fuel cells, batteries, supercapacitors, energy harvesting systems, electric and magnetic parts, conductive wires, and wearable electronic textiles. Furthermore, properties of ceramic nanofibers, which enable the above applications, and techniques to characterize them are briefly outlined.

## 1. Introduction

Ceramics are widely used in many applications due to their chemical and thermal stability, and high mechanical and electrical properties arising as a result of ionic and covalent bonds between the atoms composing them [1,2]. Recently, ceramic fibers have been developed for many advanced materials industries due to the unique properties known only in ceramic materials—superior high oxidation and corrosion resistance, semiconducting, sensibility, electric charge storage, catalytic behavior, magnetic properties, reconstruction of crystal units, tailored phase transformation, surface modification and wide range of bio-compatibility, to mention just a few [3,4,5]. 

**Figure 1:**
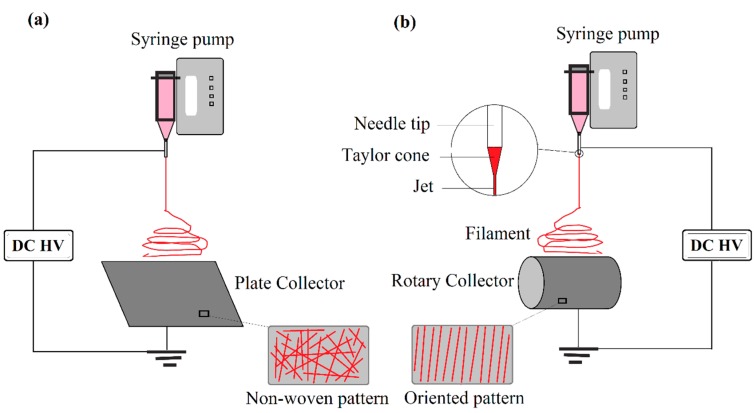
Schematic electrospinning methods.

The development of nanotechnology leads to advances in materials and creates innovative solutions to the drawbacks related to the bulk materials. With the decrease in the diameter of fibers that is required to make nanofibers, the physiochemical and structural properties of materials are modified according to the corresponding bulk materials. Several methods have been developed to fabricate NFs, such as template method [6], self-assembly [7], phase separation [8], melt blowing [9], drawing [10] and electrospinning method [11]. Among them, electrospinning is a straightforward, cost-effective, and versatile technique that essentially employs a simple and economical setup to produce NFs in a variety of shapes and sizes. For example, typical electrospinning set ups for production of random (non-woven) and aligned (oriented) NFs are shown in Figure 1. In this method, a polymer solution or melt is charged by an electric force and deformed into a cone called a Taylor cone, when electrostatic force overcomes the surface tension and viscosity of a polymer droplet. The high voltage between the needle tip with an aluminum collector causes the jet to stretch into a finer filament with evaporation of solvents. Filaments are eventually deposited on a plate or rotary collator to produce randomly non-woven or oriented NFs, respectively. They are synonymously called fibrous mat, membrane or scaffold (see Figure 1).

Electrospinning has been regarded as the most promising approach to produce continuous NFs and the fiber diameter can be adjusted from micrometers to nanometers [4]. Electrospinning is a beneficial method to synthesize NFs of single and composite phases. Moreover, electrospinning has been applied to natural and synthetic polymers, carbons and ceramics. Fibers with complex architectures, such as ribbon-shaped, porous, core-shell, or hollow can be produced by electrospinning methods. It is also possible to produce nanofibrous membranes with designed aggregate structure including alignment, patterning, and two and three-dimensional nanonets [12]. In recent years, considerable efforts have been undertaken for fabrication of ceramic NFs via electrospinning methods. Electrospun ceramic NFs are a specific classification of materials due to the morphology, microstructure, composition and properties which enable them to be used in diverse applications such as life science and health-care sectors, energy and environmental ones, agriculture and food, electronic and magnetic devices [13].

**Figure 2:**
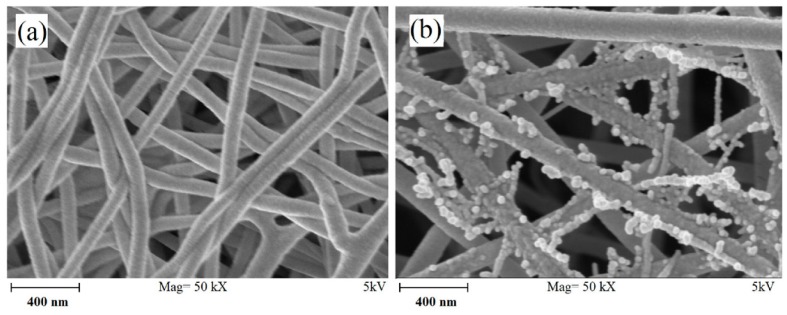
FESEM images of (**a**) electrospun PA6 nanofibers (NFs); and (**b**) decorated by hydroxyapatite (HA) nanoparticles via electrospinning method. Reprinted with permission from Ref. [16]. Copyright © 2015 Published by Elsevier B.V.

Electrospun ceramic NFs have shown many unique characteristics and have enormous application potential in widely diverse areas. Considerable researches have been conducted on exploring the properties and applications of electrospun ceramic NFs. For example, flexible electrospun TiO_2_-SiO_2_ mats are capable to curve to 1.3–3.4 mm radius of curvature even after heat treatment, while bulk ceramics are known to be brittle [14]. In another research, it is demonstrated that composite laminate electrospun mats increase the delamination strength, a great idea for using electrospun mats in industrial bonding [15]. Hybrid ceramic/polymer fibers can be easily synthesized by electrospinning method. Advantages of electrospinning methods aid to fabricate composite NFs in which the ceramic nanoparticles (NPs), hydroxyapatite (HA) here, is randomly decorated on the PA6 fibers without agglomeration (see Figure 2) [16].

This review focuses on the recent progress in electrospun ceramic NFs. Type of ceramic NFs, synthetic procedures, effective parameters to obtain ceramic NFs, surface modification and their applications are discussed with regard to the experimental findings. This review also covers special methods developed to characterize the mechanical, physical and electrical properties of electrospun ceramic NFs.

## 2. Types of Electrospun Ceramic Fibers

**Figure 3:**
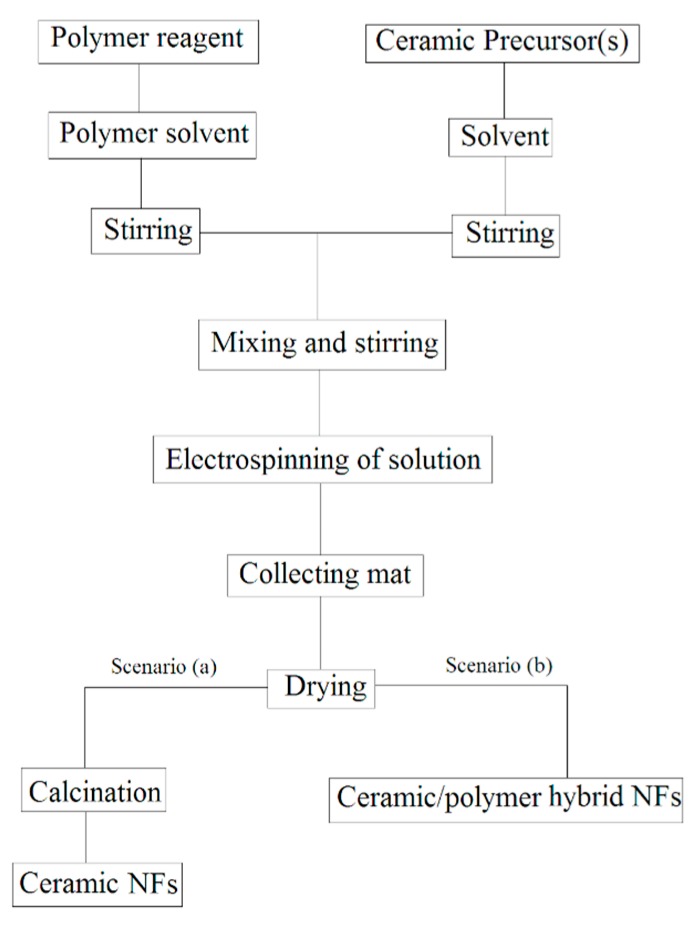
Flowchart of ceramic and ceramic/polymer NFs fabrication via electrospinning method.

As ceramic precursor solution does not have enough viscosity to make a jet during electrospinning, several successful methods have been developed to overcome this problem. Using a polymer reagent in spinning solution is the most successful method. Further, methods such as sol-gel route [17,18], which includes a polymerization stage, is an alternative for polymer reagents in the production of ceramic NFs via electrospinning. Ceramic elements are added to a polymer solution as ceramic NPs or as ceramic precursors. Viscose polymer solution that has the potential to combine one or more ceramic elements in one solution eventually results in production of single phase or composite ceramic NFs. In spite of kind of ceramic elements in solution, two scenarios can be assumed for collected NFs in mats after electrospinning (see Figure 3);

(a) Single phase ceramic NFs are obtained by elimination of polymer reagent via a certain heat treatment procedure,
(b) Ceramic/polymer hybrid NFs are synthesized without any more heat treatment.

**Figure 4:**
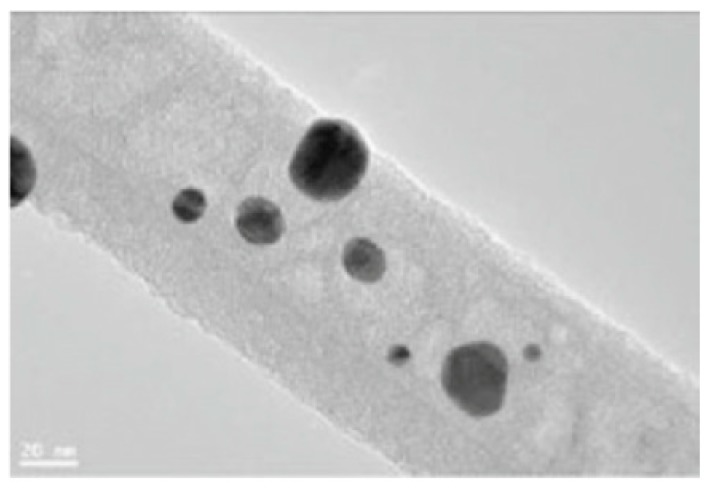
TEM images of Cu NPs inside a PAN NF. Reprinted with permission from Ref. [20]. Copyright © The Author(s) 2011.

Ceramic NPs inside the electrospun NFs will be sintered together like fibers shape or be decorated on polymer NFs following first and second scenarios, respectively. It is worth mentioning that not only ceramic NPs are used inside the polymer NFs but also metallic NPs are assigned inside the polymer NFs for desired applications. For example, Pt, Cu and Sn NPs are added in the fabrication of PVP/metal NFs for direct ethanol protonic ceramic fuel cell application [19], or for micro surface-mounted components [20]. Similar to the ceramic NFs fabrication procedure, metal NFs can be obtained by heat treatment but at lower temperatures. Pt, Sn, and Cu, metal NFs are obtained by calcination of as-spun fibrous mats at 300–450 °C after evaporation of polymer reagents [19,20] (See Figure 4).

It is worth mentioning that amorphous to highly crystalized ceramic NFs can be obtained according to the heat treatment procedure. In following sections recent developments on monolithic (single phase) and composite (hybrid) ceramic NFs are reviewed.

### 2.1. Single Phase Ceramic Fibers

Many ceramic fibers have been so far prepared by electrospinning method. Not only simple oxide ceramic fibers such as TiO_2_, Al_2_O_3_ and ZnO, but also complex oxide ceramic fibers such as CaCu_3_Ti_4_O_12_ and Li_1.6_Al_0.6_MnO_4_ have been synthesized by electrospinning. In addition, non-oxide ceramic fibers such as ZrC and Cu_2_ZnSnS_4_ are also synthesized via electrospinning method. Table 1 lists several recent simple and complex oxide and non-oxide ceramic NFs produced by electrospinning.

Ceramic precursor is often used in the form of acetate, nitrate and carbonate, using the single phase and composite ceramic NFs that could be synthesized via processing appropriate solutions. Doping of a cation inside the crystal lattice of ceramic (e.g., Mg^2+^ into hexagonal ZnO) is another advantage of this solution based route. Deionized water, ethanol, methanol or their combination are applied for solving the ceramic precursor(s). Polyvinylpyrrolidone (PVP), polyvinylacetate (PVA), etc. are common polymer reagents that dissolves in many basic or acidic solvents. Dimethylformamide (DMF) and chloroform (CF) are sometimes added to polymer solution for better charge polarization. Stirring is carried out until a homogenous and clear solution obtained. This stage could be done in short period of 1 h or prolonged to 24 h. Adjustment of pH has significant role in dissolving ceramic and polymer precursors. As mentioned above, polymer concentration controls the viscosity of solution. Extra amounts of polymer not only result in thicker fibers but also causes the destruction of ceramic fibers and elimination of polymer during calcination. Heat treatment called calcination is preformed based on the nature of ceramic, for example, ZnO NFs are synthesized by calcination at 500 °C for 2 h [21]. Calcination conditions and their effects based on the real experience are discussed in a separate Section 3.3. As can be seen in Table 1, the final morphology of ceramic NFs could differ based on the choice of polymers, spinning and calcination conditions, such as straight, smooth, tubular, hollow, shorten fiber, sintered particles and irregular shapes.

### 2.2. Composite Ceramic/Polymer Fibers

The ceramic/polymer electrospun composite NFs exhibit high surface-to-volume ratios with unique structure controlled morphologies. The inherent properties of these nanostructured fibrous materials make them suitable candidates for various advanced applications. For example, polymer/silicate NFs are used in diverse applications from biomedical, carbon fiber fabrication, food packing, to sensing [82]. Promising approaches to constructing biodegradable polymers and bioactive ceramics have been implemented via electrospinning of hybrid scaffolds [83]. A list of recent products of hybrid electrospun NFs composed by polymer matrix and ceramic NPs and their applications are given in Table 2.

## 3. Fabrication of Electrospun Ceramic Mats

### 3.1. Assistant of Polymer 

**Figure 5:**
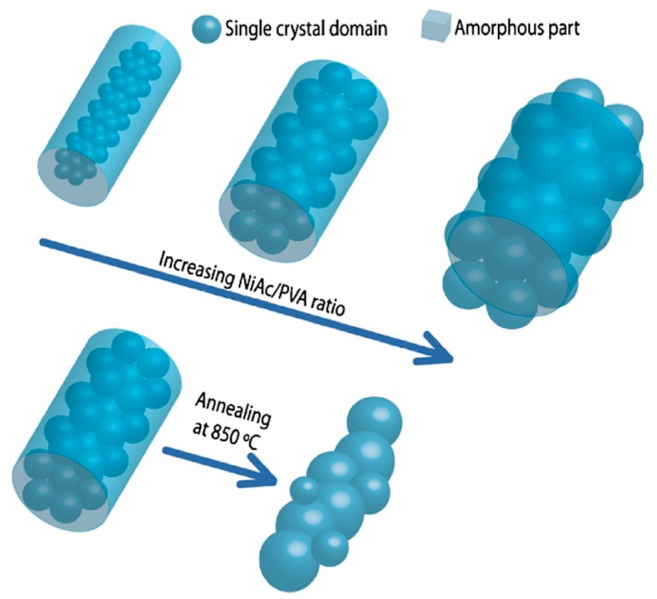
Schematic illustration of microstructural evolution in NiO NFs as a function of NiAc/PVA ratio and high temperature calcination. Reprinted with permission from Ref. [99]. Copyright © 2009 Elsevier Ltd. and Techna Group S.r.l.

Since ceramic precursor solution does not have enough viscosity to make a jet during electrospinning procedure, a polymer reagent is often used in spinning solution aimed at developing ceramic NFs. Mohammadi et al. [97] and Zadeh et al. [49] explained the role of polymer reagent in viscosity of the spinning solution of CaCu_3_Ti_4_O_12_ and mullite, respectively. Continuous electrospinning is carried out when the viscosity of electrospinning solution is optimized. Higher polymer content tends to increase the viscosity of the solution, eventually resulting in flat ribbon shaped ceramic fibers. Another role of polymer solution is to obtain fibers with different diameter and crystallinity. For example, a different mass ratio of zinc acetate/PVA (1:3, 2:3 and 1:1) causes increasing fiber diameter and crystallite size of ZnO [98]. Similar results have been obtained for NiO NFs [52,99]. Figure 5 presents how the diameter and the crystallinity of NiO NFs increase with increasing the ratio of nickel acetate/PVA in precursor.

According to Table 1, PVP and PVA are the most commonly employed polymer to synthesize ceramic single phase NFs. This is because they have high solubility in a variety of solvents and good compatibility with many metal alkoxides. To produce ceramic fibers, first soluble salts of metal are dissolved in water or ethanol and then added to polymeric solution. Polymeric solution is often prepared by adding DMF and CF in order to adjust the resultant fiber diameter and to prevent bead forming [57]. Co-solutions consist of optimized ratio of ceramic precursor with polymer reagent are mixed and then electrospun. After drying, calcination is the main step to produce ceramic single phase NFs. Recent findings indicate that mixing of PVP with PVA help to achieve ultrathin NFs. Saleemi et al. [100] synthesized magnesium cobaltite NFs using PVP, PVA and PVP/PVA (3:1) and observed that the average diameter of NFs decreased from 250 to 200 nm in the case of combined polymers. Average diameter is also decreased by using other polymer reagents such as polyethylene oxide (PEO). Starbova et al. [101] examined PEO in the fabrication of electrospun ZnO NFs and found more efficient viscous–elastic behavior of the high molar mass PEO under electro-hydrodynamic conditions compared to that of PVA. Finally, it is worth mentioning that there are many attempts to eliminate polymer reagents by using the sol-gel method. Chen et al. [17] produced mullite NFs in the absence of polymers. Essential viscosity required for electrospinning is achieved via controlling the hydrolysis step of sol gel.

### 3.2. Electrospinning Parameters and Procedures

There are many parameters that affect electrospun ceramic NFs morphology. Among them, the amount and composition of polymer in the solution are the most significant factors, as explained by many researchers [85]. However, operating device parameters also play a significant role in achieving different morphology and crystallinity of ceramic NFs. For example, shape and type of collector affect the morphology of electrospun NFs. For example, a range of needle to collector distances can be produced using a sloped collector for making multi-size fibrous mats [85]. Physical and electrical properties, adhesion and density of the NFs on substrate are also depend on the type of collector geometry. Lamastra et al. [102] examined four kinds of collectors for measuring the transmittance of NiO electrospun NFs: Al collector, sputtered Ni on quartz, and bare quartz substrate. They found that Ni-quartz target resulted in higher density of NiO NFs, while NiO-quartz target depicted more adhesion with NiO NFs. Furthermore, humidity as another aspect of environmental parameters also effect on NFs morphologies and crystallinity. Tikekar et al. [67] studied the effect of humidity (RH ~25–60%) on the microstructure of TiO_2_ electrospun NFs. They applied a heated target to form NFs at higher humidity (>60%), and observed that at higher humidity excessive plasticization of the PVP is induced and individual nanocrystals of TiO_2_ are formed.

There has been much interest in fabricating aligned NFs via electrospinning. Several methods are developed for arraying NFs in the same direction: collection of fibers across two parallel closely spaced substrates and collection of fibers by high speed rotating mandrel [103,104] are most common. Laudenslager et al. [103] reported that the parallel rotary disk (PDR) method has more advantages than the other method for aligned fiber production, besides it is the only method for fabrication of continuous NFs in diameter range of 100 to 1000 nm. Another interest in electrospinning is to fabricate twisted rope NFs. Since the NFs rope offers improved mechanical properties, these types have the potential to be used in many applications, such as artificial muscle and electronic devices. In this procedure, a tube is rotated with a motor and another is fixed to an iron support. Zheng et al. [90] successfully synthesized PVDF/CNT composite NFs rope for use in strain sensors. Synthesis of ceramic porous hollow NFs (e.g., Al_2_O_3_) is another interest in modified electrospinning devices. In this type of electrospinning device, an electrode is inserted into the PVC pipe to induce an electric charge into the solution and solution is loaded into the reservoir from which the solution flowed the pipe. The flow rate is determined by the difference of the air pressure between bottom of reservoir and inserted electrode pipe. Multiple pendent drops are formed at the holes in the pipe through changing the applied voltage and shape of the Taylor cones from which the polymeric jets are launched toward the grounded collector [105].

### 3.3. Calcination and Heat Treatment

Heat treatment of ceramic/polymer mats via electrospinning procedure has a critical role in the production of ceramic NFs. Heat treatment known as calcination, is carried out in accordance with the nature of ceramic and polymeric solution at different temperatures and soaking times. Table 1 provides a set of calcination conditions of many kinds of ceramic NFs recently fabricated. Calcination is often carried out in O_2_ atmosphere with regard to oxide ceramic fibers. However, other gases such as H_2_, N_2_ and Ar are purged into furnace to obtain non-oxide ceramic NFs. The effects of atmosphere on composition of electrospun Cu doped ZnO NFs have been investigated in two ways: first, the dried fibers are calcined at 450 °C for 3 h under flow of O_2_ and second samples calcined at 300 °C for 2.5 h in H_2_. The first procedure caused the formation of CuO, Cu_2_O, and ZnO, and the second procedure tended to in-situ reduction of CuO and Cu_2_O into Cu nanocrystals (NCs) [77]. Not only calcination is preformed to eliminate the polymer part but it is also applied to change the crystallinity of ceramic NFs. For example, ZrO_2_ have gained much attention due to their use as catalysts, thermal barrier coatings and biomaterials regarding its crystal systems. Singh et al. [79] synthesized ZrO_2_ NFs by electrospinning method and they found that calcination at different temperatures resulted in tetragonal to monoclinic phases without disrupting the fiber morphology.

**Figure 6:**
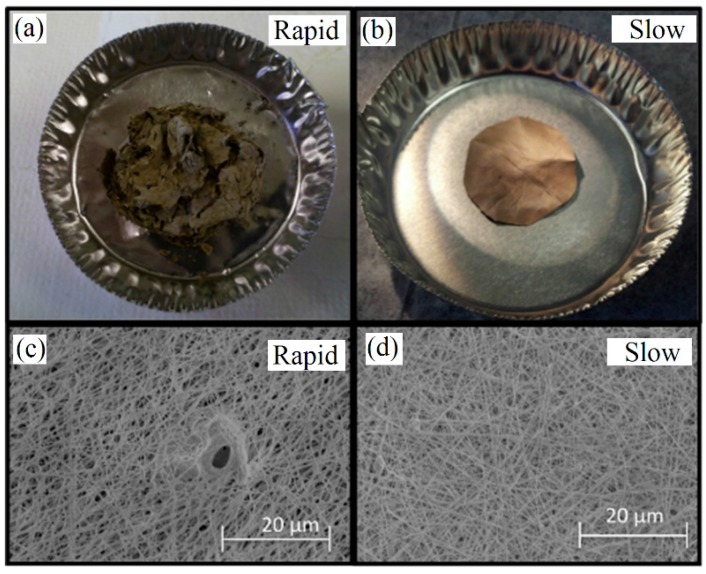
Image and SEM micrograph of (Pd/Cu) doped ceria in polyvinylpyrrolidone (PVP) matrix calcined at different heating rate; (**a**,**c**) rapid and (**b**,**d**) slow. Reprinted with permission from Ref. [41]. Copyright © 2014 Elsevier B.V.

The heat treatment parameter plays significant role in the final size and morphology of fibers. A low heating rate is often applied to ensure the removal of organic components without destroying the NFs appearance and also to avoid ceramic NFs breaking to small parts due to rather poor thermal shock resistance of ceramics [106]. Gibbons et al. [41] examined calcination of (Pd/Cu) doped CeO_2_ in PVP matrix NFs via rapid (2 K·min^−1^) and slow (0.1 K·min^−1^) heating rate. A non-woven mat of CeO_2_ based NFs with average diameter <200 nm was achieved with slow oxidative calcination. However, with rapid heating, thicker fibers and micro-defects remained in the final mat due to melting and removal of polymer (see Figure 6).

**Figure 7:**
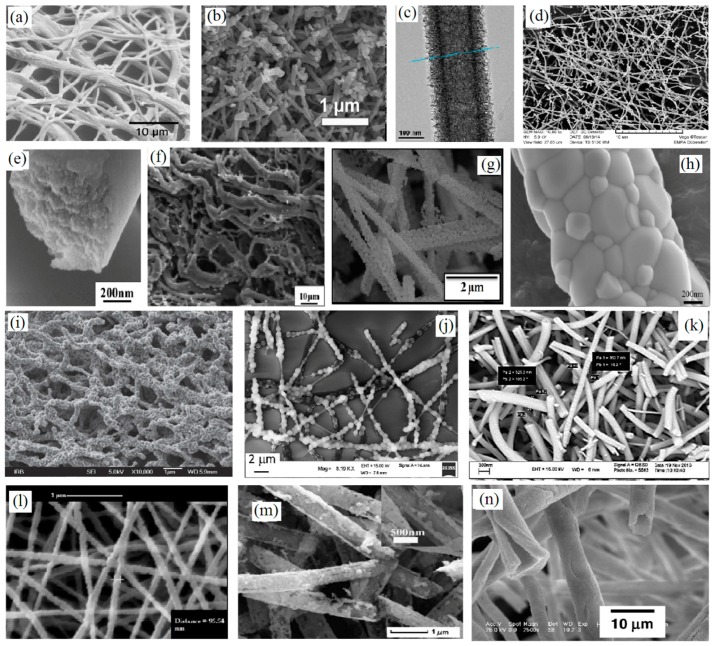
Micrographs of ceramic fibers synthesized by electrospinning method. (**a**) HA NFs calcined at 700 °C. Reprinted with permission from Ref. [40]; (**b**) Pr_0.4_Sr_0.6_Co_0.2_Fe_0.7_Nb_0.1_O_3−δ_ NFs calcined at 800 °C. Reprinted with permission from Ref. [55]; (**c**) MnO_2_ NFs calcined at 1000 °C. Reprinted with permission from Ref. [29]; (**d**) CaCu_3_Ti_4_O_12_ NFs calcined at 900 °C. Reprinted with permission from Ref. [97]; (**e**) Al_2_O_3_ NF calcined at 800 °C. Reprinted with permission from Ref. [24]; (**f**) BaFe_12_O_19_ NFs calcined at 800 °C. Reprinted with permission from Ref. [25]; (**g**) CdTiO_3_ NFs calcined at 600 °C. Reprinted with permission from Ref. [32]; (**h**) La_2_Zr_2_O_7_ NFs calcined at 1400 °C. Reprinted with permission from Ref. [46]; (**i**) NiO NFs calcined at 400 °C. Reprinted with permission from Ref. [52]; (**j**) SiO_2_ NFs calcined at 400 °C. Reprinted with permission from Ref. [56]; (**k**) TiO_2_ NFs calcined at 500 °C. Reprinted with permission from Ref. [68]; (**l**) Mullite NFs calcined at 1200 °C. Reprinted with permission from Ref. [49]; (**m**) ZrC NFs after pyrolysis at 1400 °C. Reprinted with permission from Ref. [78]; and (**n**) 8YSZ NFs calcined at 1400 °C. Reprinted with permission from Ref. [81]. [40] Copyright © 2011 Elsevier B.V. [55] Copyright © 2016 Elsevier Ltd. and Techna Group S.r.l. [29] Copyright © 2011 Elsevier Ltd. [97] Copyright & 2015 Elsevier Ltd. and Techna Group S.r.l. [24] Copyright © 2012 Elsevier B.V. [25] Copyright © 2016 Elsevier B.V. [32] Copyright © 2013 Elsevier Ltd. and Techna Group S.r.l. [46] Copyright © 2016 Elsevier Ltd. and Techna Group S.r.l. [52] Copyright © 2014 Elsevier Ltd. and Techna Group S.r.l. [56] Copyright © 2015 Elsevier Ltd. [68] Copyright © 2014 Elsevier B.V. [49] Copyright © 2013 Elsevier Ltd. and Techna Group S.r.l. [78] Copyright © 2014 Elsevier Ltd. and Techna Group S.r.l. [81] Copyright © 2007 Elsevier B.V.

**Figure 8:**
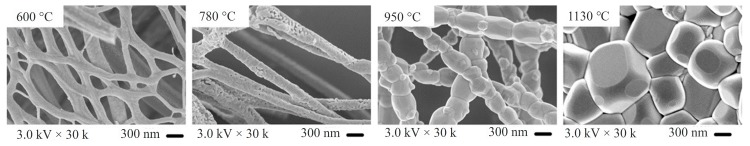
SEM images of CaCu_3_Ti_4_O_12_ composite NFs calcined at different temperatures. Reprinted with permission from Ref. [30]. Copyright © 2012 Elsevier B.V.

It is worth knowing that fiber structure is not always achieved by calcination. Our findings based on the literature review as shown in Figure 7 confirm the above statement. Smooth, straight, broken, short fiber, sintered fibers and particles, belt and ribbon, hollow and porous fibers could be the final morphologies after calcination of ceramic/polymer electrospun mats. The morphology of pristine fibers changes dramatically at different temperature of calcination depending on the polymer matrix and ceramic precursor. STA analysis of as-spun mats is usually employed to determine the optimum calcination temperature. Degradation of polymer reagent, ethanol, nitrates, carbonates and acetate groups in most as-spun mats are occurred at temperature <500 °C. A region can be observed at higher temperature in the differential scanning calorimetry-thermogravimetry (DSC-TG) curves such that no weight loss occurred after a certain temperature [31]. The final stage of calcination is performed at this temperature to form single phase ceramic fibers. Figure 8 shows the SEM images of CaCu_3_Ti_4_O_12_ composite NFs calcined at different temperatures (600 to 1130 °C) [30]. It is obvious that the morphology of the final NFs depends on the calcination temperature. Calcination at lower temperatures tends to form smooth surface NFs while higher temperatures tend to form porous ceramic NFs due to degradation of organic compounds (such as nitrates, acetates and PVP). However, further increasing of calcination temperature causes eliminating pores from surface of NFs, and suggests that coarse grained ceramics NFs are formed due to the sintering. Further heat treatment causes to NFs become a bulk ceramic.

**Figure 9:**
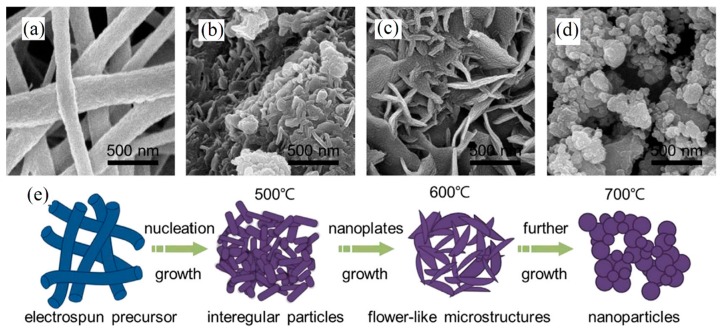
SEM image PVA/ Li_1.2_Ni_0.17_Co_0.17_Mn_0.5_O_2_ pristine NFs changes to nanoplates with an open porous structure after calcination and finally transform to flower-like microstructure at elevated temperature (**a**–**d**); and (**e**) schematic illustration of the growth mechanism of flower-like Li_1.2_Ni_0.17_Co_0.17_Mn_0.5_O_2_ microstructures. Reprinted with permission from Ref. [107]. Copyright © 2013 Elsevier Ltd. and Techna Group S.r.l.

Achievement of morphology different form original electrospun fibers has been observed by several researches. For example, flower-like Li_1.2_Ni_0.17_Co_0.17_Mn_0.5_O_2_ microstructures was the favorable morphology to facilitate the diffusion of lithium ions into pores of fabricated mat as an electrode of a battery [107]. Figure 9 shows how PVA/ Li_1.2_Ni_0.17_Co_0.17_Mn_0.5_O_2_ pristine NFs change to nanoplates (NPLs) with an open porous structure after calcination and finally transform to flower-like microstructure at elevated temperatures.

### 3.4. Surface Modification of Electrospun Ceramic Mats

Two dimensional electrospun mats have high surface area in comparison to the other forms of materials. There are many attempts to enhance the surface activity of NFs. Pyrolysis, hydrothermal, carbothermal and other process have been performed on electrospun ceramic NFs to enhance their surface activity promoting biomedical, electronic, sensor applications. 

Pyrolysis of electrospun NFs is a new and effective method to beneficially allow for large scale NF production. In this method, only the pyrolysis step required to transform the polymers to ceramics at lower temperatures. This method is faster than common electrospun ceramic NF processing which has multistep production and needs elevated temperature [108]. By pyrolysis of electrospun NFs at lower temperature not only oxide ceramic NFs can be produced but also non-oxide ceramic NFs (e.g., SiC, Si_3_N_4_, TiC) can be synthesized. SiO_2_, Si_3_N_4_ and Si_2_N_2_O nanowires (NWs) are achieved from electrospinning mats afterwards by pyrolysis under N_2_ flow at 1300 °C for 2 h. TiC NFs are also synthesized first by electrospinning of polyacrylonitrile and titanium isopropoxide solution and then thermally stabilized at 270 °C in air for 3 h and then carbonized and pyrolyzed under Ar at 1000 °C for 3 h [109]. The hierarchical structure including fibers decorated with SiO_2_, Si_3_N_4_ and Si_2_N_2_O NWs possess a higher specific surface than simple NFs, which is more beneficial in gas sensor devices [110]. Although microstructure NFs can be exchanged to desirable shape via pyrolysis method, there are some problems to obtain perfect NFs. Eick et al. [108] overcame breaking the fibers during pyrolysis by means of UV irradiation on mats that crosslinks the polymer and prevents fibers to flow during pyrolysis.

**Figure 10:**
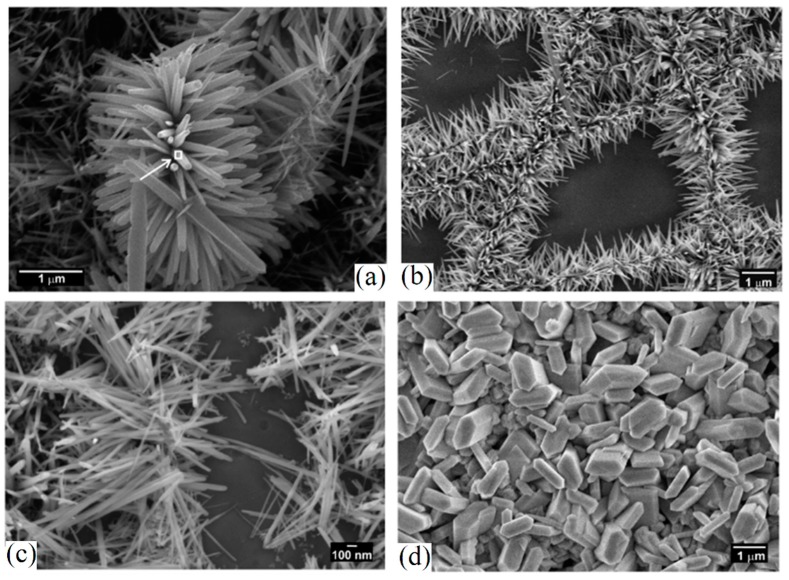
FESEM micrographs of hydrothermal process carried out on ZnO NFs at different conditions; (**a**) 1 min electrospinning and 1 h autoclaving (**b**) 2 min electrospinning and 2 h autoclaving (**c**) 10 min electrospinning and 2 h autoclaving (**d**) 1 min electrospinning and 8 h autoclaving. Reprinted with permission from Ref. [111]. Copyright © 2016 Elsevier B.V.

The hydrothermal process is an effective method to improve surface activity of electrospun ceramic NFs. Figure 10 shows the effect of the hydrothermal process on ZnO NFs at 160 °C in aqueous medium containing hydrolyzed zinc acetylacetonate at different conditions [111]. Time of hydrothermal processing affects the recrystallization and morphology of ZnO NFs. This is because of the adsorption of zinc hydrolytical products as well as an acetylacetonate group on selected crystal planes.

Carbothermal reduction is employed to achieve hollow fibers. Generally, carbothermal reduction is carried out in two steps: the first step at lower temperature in vacuum and the second step at elevated temperature in N_2_ atmosphere. AlN and ZrN hollow NFs are synthesized by electrospinning of common precursor following carbothermal reduction. By this method, rough and hexagonal crystal of AlN and ZrN are formed on the surface of NFs. The outer diameter and thickness of hollow fibers are 500 and 100 nm, respectively [112,113].

**Figure 11:**
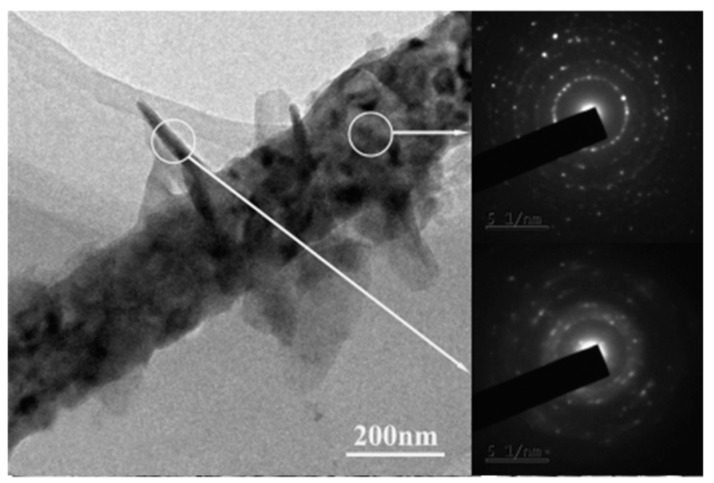
TEM image and electron diffraction patterns of selected areas of a TiO_2_/V_2_O_5_ composite NFs. Reprinted with permission from Ref. [66]. Copyright © 2014 Elsevier Ltd. and Techna Group S.r.l.

Surface modification of ceramic NFs has been achieved chemically using organic and inorganic solutions. In this case, ceramic or polymer/ceramic hybrid NFs are treated using an appropriate sol and subjected to controlled calcination in accordance with the nature of the secondary phase. Branched NFs of TiO_2_ NFs are achieved via immersing in V_2_O_5_ sol and subsequent calcination at 550 °C for 2 h at N_2_ atmosphere (see Figure 11) [66]. Qin et al. [114] found that soaking ceramic NFs in water or air before or after calcination is also useful to change their microstructure. Functionalization is a practical method for synthesizing hollow NFs. Huang et al. [115] functionalized the SiO_2_ electrospun NFs and found that silica shell is covalently decorated on the hybrid fiber surface by hydrolysis and condensation of silyl functional groups with the tetraethoxyorthosilane (TEOS) in an ethanolic ammonia suspension. After thermal decomposition of the polymeric fiber templates, inorganic silica hollow fibers are formed that mimic the structure and morphology of the fiber templates.

**Figure 12:**
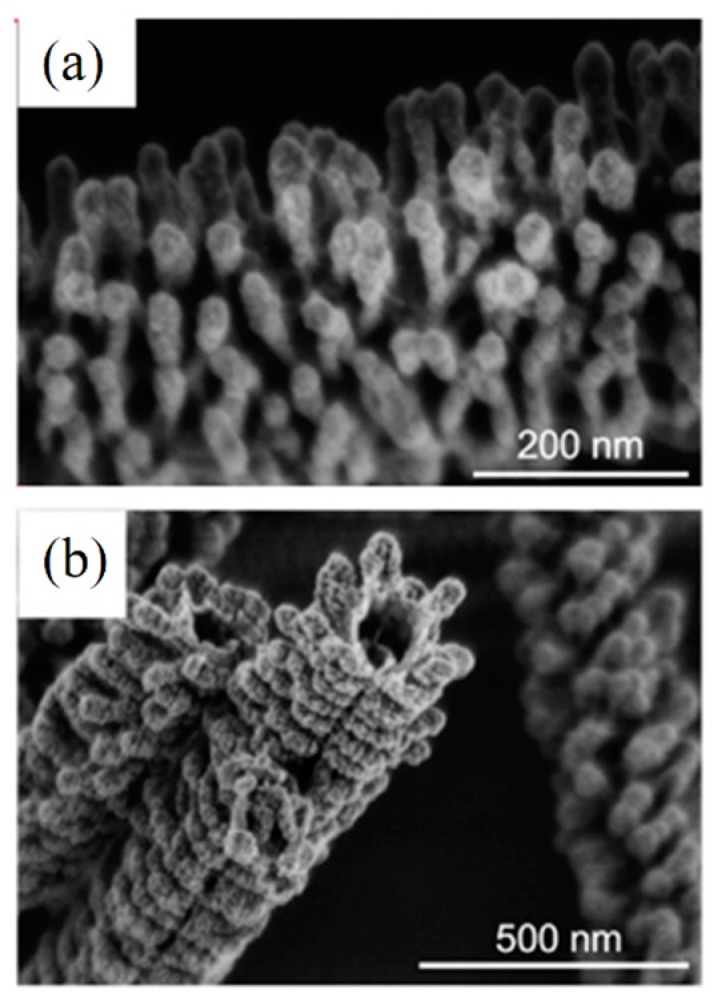
SEM micrographs of SnO_2_ NFs. (**a**) plasma etching time of 30 s and sputtering time of 190 s; and (**b**) plasma etching time of 30 s and sputtering time of 480 s. Reprinted with permission from Ref. [116]. Copyright © 2016 Elsevier Ltd.

Plasma etching, sputtering and annealing of electrospun NFs can make the high surface area for electrospun NFs. SEM micrographs of SnO_2_ NFs modified by plasma etching and sputtering process at different conditions to achieve hierarchical NFs are presented in Figure 12 [116].

## 4. Characterization of Ceramic Electrospun Mats 

The properties of electrospun NFs can be studied throughout different techniques. Microstructural features, mechanical, physical and electrical properties of ceramic NFs are different from those of bulk materials. Hence, the characterizations of electrospun NFs are discussed below.

### 4.1. Microstructure 

**Figure 13:**
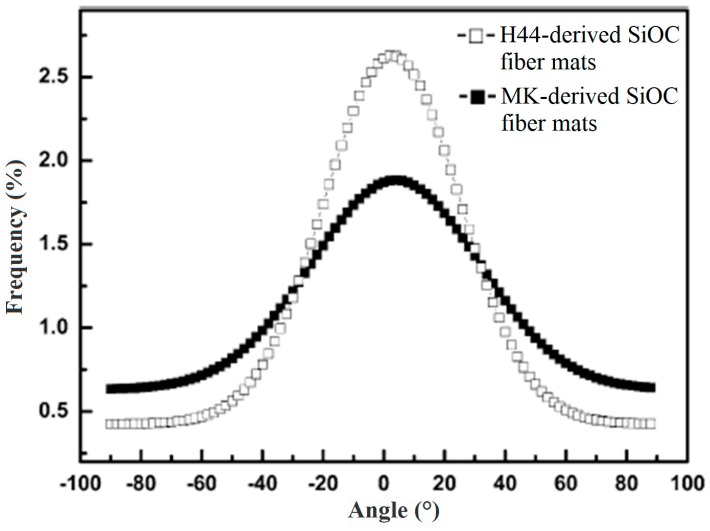
A typical directionality (angle distribution) of SiOC Nfs prepared with different precursors. Reprinted with permission from Ref. [57]. Copyright © Springer Science+Business Media New York 2015.

Electrospun NFs can either be randomly oriented in the length and width directions or in contrast be aligned unidirectionally in the plane of the mat. Directionality (angle distribution) of fibers histogram can be useful for studying the arrangement of fibers. For this matter, the number of fibers in each orientation is measured and then frequency versus angle is plotted in a graph. A typical angle distribution of SiOC fibers produced from different precursors is presented in Figure 13 [57]. It is worth explaining that flat histograms represent the random oriented NFs, and histograms with a narrow and sharp peak present a preferred orientation. Furthermore, two peaks histograms demonstrate cross linked oriented fibers. Therefore, histograms shown in Figure 13 indicate both kinds of fibers have a preferred orientation.

### 4.2. Mechanical Properties

The tensile strength of fibrous electrospun mats are measured using an electro-force planar biaxial test bench instrument via applying uniaxial stress in accordance to ISO 527-3 standard. Stretching rate and shape of the test sample are two critical factors to achieve accurate results. According to the displacement of fixture and loading force, a stress-strain curve is plotted to study the mechanical properties of mats such as elastic and plastic stage, Young module as well as ultimate tensile strength (UTS).

**Figure 14:**
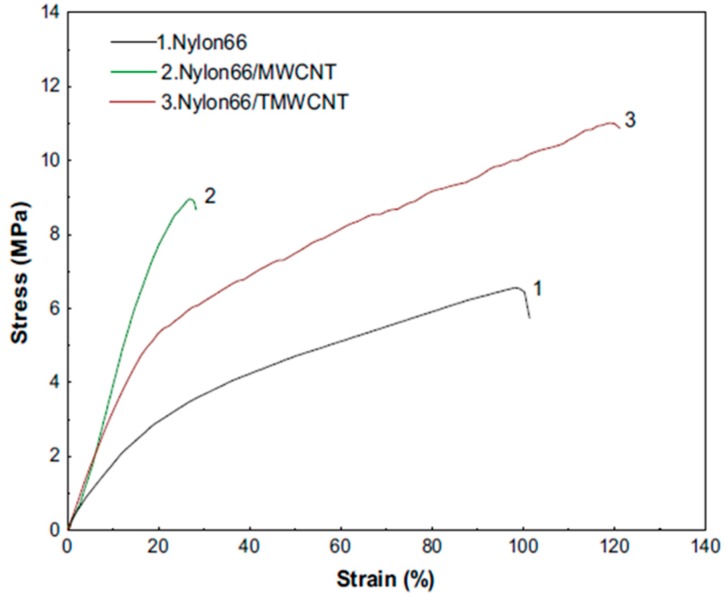
Strain–stress curves of PA66, PA66/ MWCNT and PA66/ TMWCNT NFs. Reprinted with permission from Ref. [118]. Copyright © 2013 Elsevier Ltd. and Techna Group S.r.l.

The strain–stress curves of the PA66, PA66/MWCNT (multiwalled carbon nanotube) and PA66/TMWCNT (treated multiwalled carbon nanotube) are shown in Figure 14. As can be seen, MWCNT-based polymer composites have better mechanical properties than pristine polymers owing to the reinforcing effect of MWCNT. Mixing of CNT and MWCNT into polymer NFs causes a significant enhancement of mechanical properties due to enhancement of β phase and elasticity, and also formation of a stable three-dimensional conducting network [117,118]. Xiang et al. [119] have also investigated the incorporation of CNT NFs inside the electrospun PA6 fibrous mats, and found that fiber-fiber load sharing can be enhanced by using each following methods; increasing friction between fibers, thermal bonding, and solvent bonding. Moreover, adding the NPs into polymer NFs usually has similar results. Al_2_O_3_ and TiO_2_ NPs as typical ceramic NPs modify the roughness of the fibers and affect the interfacial adhesion between the filler and the polymer matrix. Although Young’s modulus and tensile strength were improved with addition of NPs, a less pronounced effect was found for ductility and stiffness of electrospun mats [120,121]. It is worth mentioning that using metallic cations (e.g., Fe^3+^) inside the polymer solution reinforces the fibers due to changing the pH, functionalization and enhancement of organic group attachments [122]. 

Eventually it is worth mentioning that in some applications such as water filtration and tissue engineering, tensile strength is measured via two type methods; dry and wet conditions [123]. The wet condition is carried out in the same way as the dry but firstly samples are immersed in a water or a bio-solution container for a certain period of time, and then pulled out and quickly examined by the above procedure.

### 4.3. Physical Properties

#### 4.3.1. Porosity

The total porosity of electrospun fibrous mats can be measured with different methods. Bulk density method, mercury intrusion porosimetry (MIP), X-ray computed tomography (X-CT), and Barrett-Joyner-Halenda (BJH) analysis are practical methods for determination of pore size and pore size distribution of electrospun ceramics mats.

(a) Bulk Density Method

The total porosity is calculated based on the following equation;

(1) $$Porosity(\%)=100-\frac{\rho_{0}}{\rho }$$

where *ρ* and *ρ*_0_ are bulk and true density, respectively. Bulk density is calculated by dividing the weight by the volume of mat, and true density is measured by gas pycnometry method [57]. 

(b) Mercury Intrusion Porosimetry (MIP)

In this method, the pore size is measured in accordance with the external pressure needed to force the liquid into a pore against the opposing force of the surface tension of the liquid [57]. The basic formula used in this method is:

(2) $$Porosity(\%)=\frac{V_{Pore}}{V_{Pore}+V_{Apparent}}\times 100$$

where, *V_Pore_* is the total pore volume of the test sample, and *V_Apparent_* is the apparent volume of the test sample. For this technique, a porosimeter device is used for the analysis of pore structure of fibrous mats.

(c) X-ray Computed Tomography (X-CT) 

This method is a NDT technique for preparing digital data of samples like those in electrospun fibrous mats by using computer processed X-ray to produce slices of specific areas of the body. Then a three-dimensional image is built by stacking a large series of axial slice and carried out for calculating solid fraction (*SF*). The porosity is then calculated via the following equation [124];

(3) Porosity(%)=100%−SF%

(d) Barrett-Joyner-Halenda (BJH)

BJH analysis is an analytical method to measure the pore size distribution of mesoporous materials. Electrospun ceramic mats (e.g., Zn_2_SnO_4_) can be easily characterized by this method [125]. In this method, the amount of gas, preferably nitrogen, desorbed on the sample as a function of the partial gas pressure is measured at 77 K. The modified Kelvin Equation (4) is then used to relate the amount of adsorbate removed from the pores of the material, as the relative pressure (*P*/*P*_0_) is decreased from unity to a lower value, to the size of the pores [126].

(4) $$r_{k}=-\frac{2\gamma V}{RTLn(P/P_{0})}$$

where *r_k_* is Kelvin radius; *V* the mole volume of nitrogen; and *γ* the surface tension of liquid nitrogen.

#### 4.3.2. Gas Permeability

Gas permeability of electrospun fibrous mats are measured under inert gas flow (e.g., N_2_ or Ar) on a disk with a certain diameter, mostly ~35 mm. A device calculates the permeability constant and uses Forchheimer’s equation as follows [57];

(5) $$\frac{P_{a}^{2}-P_{b}^{2}}{2P_{b}L}=\frac{\mu }{k}\nu_{s}$$

where *P_a_* and *P_b_* are the absolute gas pressures at the entrance and exit of the sample, respectively. *v_s_* and *L* are the superficial fluid velocity and sample thickness, respectively. *F* and *µ* are gas density and viscosity, respectively.

#### 4.3.3. Water Permeability

The water permeability test is performed using a dead-end filtration cell with a certain thickness of membrane and filtration area. Before water permeability test, usually membranes are immersed in ethanol for 1 h, and then, the membranes are sufficiently washed with de-ionized water. The deionized water is filled in a reservoir and the filtration pressure is maintained by N_2_ or Ar gas. The weight of the permeated water is measured for a certain period of time and applying pressure, and the water permeability is calculated by Equation (6) [127]:

(6) $$\mathrm{Water}\;\mathrm{permeabilty}\;=\frac{m}{\mathrm{tAP}}$$

where m is the mass of the permeated water (kg), t is the sampling time (s), A is the effective membrane area (m^2^), and P is the pressure (bar).

#### 4.3.4. Turbidity 

The turbidity test is performed to observe the rejection of particulates and changes in the turbidity. The certain amount of target solution is prepared and the test is performed using a dead-end filtration system at room temperature as well as at certain pressure. According to the turbidity of the samples, rejection rate is calculated using the following equation:

(7) $$\mathrm{Rejection}\;\mathrm{rate}\;(\%)\;=(1-\frac{C_{f}}{C_{i}})\times 100$$

where *C_i_* is the initial and *C_f_* is the concentration of permeate. *C_i_* and *C_f_* can be calculated by UV-Vis technique or using a turbidimeter [127].

#### 4.3.5. Thermal Conductivity 

Thermal conductivity has an important role during calcination of ceramic NFs. Fast weight loss of polymeric compound creates more pores which are reasons for decreased thermal conductivity. Phonon scattering centers and the phonon thermal conductivity depend on the concentration of defects. By decreasing the grain size of the polycrystalline sample, the defects increase which provide effective phonon scattering centers and thus reduce the phonon thermal conductivity. In addition, the presence of porosity also has large effects in decreasing the thermal conductivity of a solid [46]. The thermal conductivity of a polycrystalline ceramic NFs (e.g., La_2_Zr_2_O_7_) can be calculated by the following equation [128]. This equation is valid for temperatures lower than 800 °C.

(8) $$\kappa =C_{v}\nu_{m}\Lambda /3$$

where *C_v_* is the specific heat, *ν_m_* is the speed of sound and Ʌ is the phonon mean-free path. 

#### 4.3.6. Gas Sensing 

Gas sensing test is carried out generally by mounting the interdigitated electrodes (IDEs) in a quartz tube placed inside furnace. The IDEs are connected to a resistance monitoring setup via platinum wires. The cyclic exposure of the sensors to the analyte gases (e.g., H_2_ and NH_3_) is achieved with the aid of mass flow controllers. The total gas flow rate is maintained constant during the sensing test, which is carried out at a desired temperature. For ensuring stable resistance, the sensor is equilibrated in dry air overnight at the required temperature before beginning of the gas sensing experiments. During equilibration, dry air is flowed at a constant rate (e.g., 200 cm^3^/min) and sensor signal, which represents the magnitude of the change in electrical resistance when exposed to analyte gas, is defined using the following equation [99];

(9) $$\frac{dR}{R}=\frac{R_{gas}-R_{air}}{R_{air}}$$

where *R_gas_* and *R_air_* represent the measured resistances when the sensors are exposed to the analyte gas and air, respectively.

#### 4.3.7. Hydrophobicity

To investigate the hydrophilic or hydrophobic properties of electrospun mats, contact angle of a liquid on its surface is measured. For this technique, water, ethanol or their mixture is used to measure the contact angle. It is worth knowing that the surface tension of the mixture solution decreases with adding the ethanol to water. The height (*y*) and the half width (*x*) of the formed droplet on the target surface are measured to calculate the contact angle (*θ*) using the following equation [129]:

(10) $$\cos\theta =\frac{x^{2}-y^{2}}{x^{2}+y^{2}}$$

It is worth mentioning that the electrospun fiber mats are capable to be superhydrophobic, hydrophobic and hydrophilic. There are some critical parameters affecting the water contact angle (WCA) values: porosity, pore size, pore size distribution and surface roughness that depend on morphology of electrospun fibers. Pore size and surface roughness also depend on fiber diameter. Cho et al. [129] showed that the porosity sharply increases as the fiber diameter increases and reaches a plateau after a critical fiber diameter. The fiber mats with a large deviation of fiber diameter and high surface roughness show a large change of the contact angle. Furthermore, single phase electrospun polymer NFs are superhydrophobic and hydrophobic. By adding the ceramic NPs to polymer NFs, WCA decreased and hydrophilic surface are formed with regard to the nature of molecular groups of ceramic (e.g., nitride, oxide, hydroxyl, phosphate) [130]. 

#### 4.3.8. Zeta Potential 

**Figure 15:**
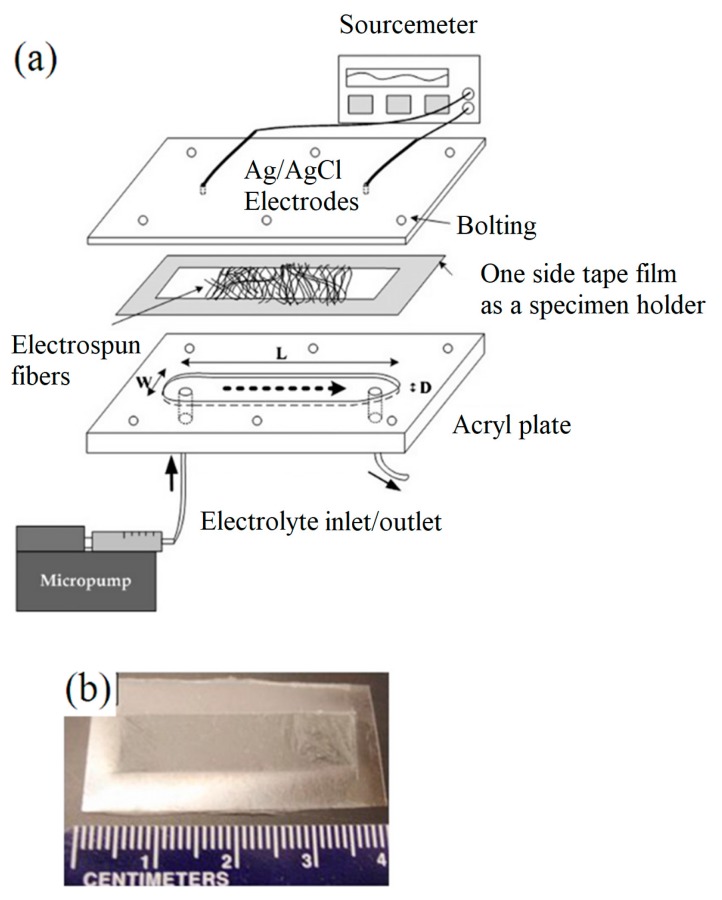
(**a**) Schematic of the microfluidic channel device for Zeta potential measurement of electrospun NFs (**b**) digital camera image of electrospun NF specimen. Reprinted with permission from Ref. [131]. Copyright © 2012 Elsevier Inc.

The zeta potential measurement of electrospun NFs mats are different from powder samples that require specimen holder preparation prior to use a commercial Zetasizer. For this technique, first two acrylic plates are machined and assembled to form a microfluidic channel (150 µm high, 2.0 mm wide, and 30 mm long). A frame is formed outside the hole where electrospun NFs are spun to cover around the frame (see Figure 15). Two electrodes for the measurement of streaming currents are housed in the top plate. A programmable micropump is used to apply fluid pressure with controlled flow rate (0.1 to 1.6 mL·min^−1^). Different pH buffer solutions can be used in this method in order to characterize the zeta potentials [131].

### 4.4. Electrical Properties

#### 4.4.1. Dielectric Constant

There is no direct method to measure the dielectric constant of NFs because the dimensions of NFs are much smaller than those required for standard measurements. Another problem occurring during measurement of NFs is the existence of pores in the NFs mats. Researchers solved this problem by applying the mixture rule as shown in Equation (11) [30].

(11) $$\log\varepsilon_{c}=\nu_{1}\times \log\varepsilon_{1}+\nu_{2}\times \log\varepsilon_{2}$$

where *ε_c_*, *ε*_1_ and *ε*_2_ stand for the dielectric constant of a ceramic/polymer composite, polymer, and ceramic, respectively; *ν*_1_ and *ν*_2_ represent the volume fraction of the polymer and ceramic, respectively. 

#### 4.4.2. Electrolyte Uptake 

In order to measure electrolyte uptake and ionic conductivity of electrospun mats which are important in many applications, the mat is immersed in liquid electrolyte for a period of time. After immersion, the membrane is taken out of the electrolyte solution and the excess electrolyte solution on the surface of the separator is wiped off with filter paper. The uptake of electrolyte solution is determined using the following equation [86,132];

(12) $$Uptake(\%)=\frac{W-W_{0}}{W_{0}}\times 100$$

where *W*_0_ and *W* are the weights of the electrospun mat before and after soaking in the liquid electrolyte, respectively.

#### 4.4.3. Ionic Conductivity

AC impedance measurements using an impedance analyzer over the variable frequency ranges and amplitude are performed to measure the ionic conductivity and interfacial resistance of nanofibrous mats [86]. The following procedure is used to measure ionic conductivity of electrospun mats. First the electrolyte sample is sandwiched between two stainless steel electrodes and the impedance measurements are performed at certain amplitude over the desired frequency range. The cell is kept for some time (e.g., 5 h) to ensure thermal equilibration of the sample before measurement. The interfacial resistance R_f_ between the polymer electrolyte and lithium metal electrode is measured at room temperature by the impedance response of Li/polymer electrolyte/Li cells over the frequency range 10 mHz to 2 MHz at an amplitude of 20 mV. The electrochemical stability is determined by linear sweep voltammetry (LSV) of Li/polymer electrolyte/steel cells at a scan rate of 1 mV/s over the range of 2–5.5 V at 25 °C [132].

#### 4.4.4. Battery Efficiency 

The following procedure uses the battery test of electrospun mats. Two-electrode lithium prototype coin cells are fabricated by placing the electrospun polymer electrolyte between lithium metal anode and carbon coated lithium iron phosphate (LiFePO_4_) cathode. Then the electrochemical tests of the Li/polymer electrolyte/LiFePO_4_ cells are conducted in an automatic galvanostatic charge–discharge unit at 25 °C at a certain current density. The activation of electrospun membrane to prepare polymer electrolyte and the fabrication of test cells are carried out in an argon-filled glove box with a moisture level <10 ppm [132].

#### 4.4.5. Permittivity, Magnetic Permeability, and EMI Shielding Efficiency (SE)

The ASTMD-4935 standard is used for measuring the permittivity, magnetic permeability, and electromagnetic interface (EMI) shielding efficiency (SE) of two-dimensional materials like electrospun mats. In this method, a network analyzer equipped with an amplifier and a scattering parameter (S-parameter) test set over a frequency range of 800–8500 MHz. The annular disk made of electrospun mats are prepared by punching machine, and EMI shielding efficiency is calculated using the S-parameters [88,133].

#### 4.4.6. Harvest Energy Performance 

**Figure 16:**
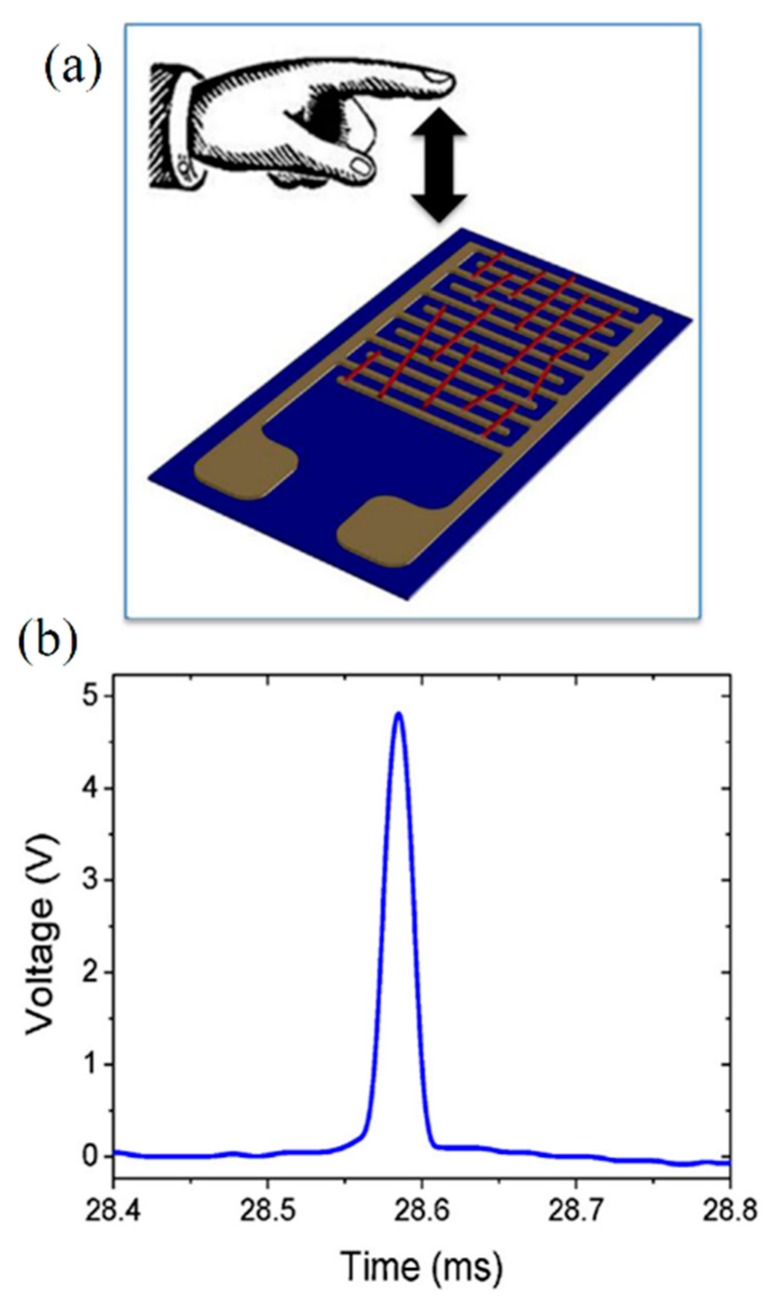
(**a**) Schematic of energy harvest examination by bending test; and (**b**) positive output voltage generated during a test performed with finger deformation for the PVDF electrospun NFs. Reprinted with permission from Ref. [93]. Copyright © 2013 Elsevier B.V.

In order to preform the bending test examination, first NFs are collected on an interdigitated electrode plates as shown in Figure 16a and then, in order to study the effect of larger deformations on the output voltage of the electroactive NFs, a finger which protected by an insulator glove in order to prevent interferences from human bioelectricity, is used to apply a periodic dynamic loading on the top of the generator by simple tapping during which, the positive and the negative output voltage is measured. According to the results obtained by Nunes-Pereira et al. [93] the highest output voltage depends on mechanical properties of NFs. Moreover, decoration of polymer NFs by ceramic NPs is not always appropriate for energy harvest application because of increases of mechanical strength.

## 5. Applications of Ceramic Electrospun Mats

Ceramic NFs have recently been recognized as advanced materials due to their special properties and microstructures [12]. In accordance with our knowledge, several applications can be assumed for ceramic NFs: catalyst, membrane, sensor, biomaterial, fuel cell, and parts of electronic device and batteries. Ceramic NFs application is not limited to the above-mentioned fields, new application areas have been introduced for using of NFs such as fire-resistant fabrics or sound adsorbent materials. Moreover, the microstructure, composition and size of NFs can be controlled via electrospinning procedure, thus high practical NFs are developed in accordance with requested applications.

### 5.1. Catalysts and Photocatalysts

Ceramic NFs are widely used in many photocatalystic applications, and fortunately they can be fabricated in different morphology such as hollow, porous, belt and solid via electrospinning procedures. Photocatalytic activity of electrospun ceramic NFs are generally carried out by using different organic targets such as methylene blue (MB), methylene orange (MO), and Rhodamine B (RhB). Choi et al. [134] demonstrated a new type of visible light-induced photocatalyst, using fluorescein molecules, TiO_2_, and gold NPs decorated on electrospun polymer NFs. It was found that the photo degradation efficiency of TiO_2_/polymer microstructure is nearly three times greater for MB than Degussa P25, which was used as a control material. Zhao et al. found that the decomposition rate of MO by branched TiO_2_/V_2_O_5_ hybrid NFs increased by ~96% relative to single phase TiO_2_ NFs. It is because of formation of V^4+^ and Ti^3+^ on the surface of NFs which have narrow band gap and lower electron-hole recombination rates [66]. In spite of the benefits of electrospun NFs, it is worth mentioning that photocatalytic activity of some ceramics (e.g., TiO_2_) prepared by hydrothermal reaction is higher than that prepared by blended spinning [135]. In accordance with the results obtained by Dong et al. [136] ZnO nanobelts (NBs) show the best photocatalytic performance for the degradation of RhB. Furthermore, it is found that the deposition of Au NPs on ZnO NBs can further enhance the photocatalytic activity owing to the formation of ohmic contact. Pascariu et al. [137] also showed that the efficiency of electrospun ZnO NFs for degradation of RhB is improved by incorporation of SnO_2_ inside the fibers for an optimum Sn/Zn molar ratio of 0.030. 

In addition, there are many studies on the photocatalysis property of electrospun NFs on actual targets. Wang et al. examined the electrospun Ni/Al_2_O_3_ NFs as a catalyst on the dry reforming of methane. They studied the effect of calcination temperature on the catalyst performance and found that the catalyst reactivity in the dry reforming of methane decreased with increasing calcination temperature. Furthermore, more and uniform Ni NPs are produced in attachment on NFs at high reduction temperatures. The reduction temperature effect is also confirmed by the reactivity during the dry reforming of methane [54]. In another work, Hassan et al. [32] explained that electrospun CdTiO_3_ NFs have the potential for the removal of pollutants and noxious wastes. They found that calcination of as-spun NFs has better results for photocatalytic activities due to higher crystallinity and a red shifted absorption wavelength.

Not only are common ceramic NFs (e.g., TiO_2_, Al_2_O_3_ and ZnO) used in photocatalysis application, but advanced ceramics are also assigned for photo- and the other catalysis applications. For example, electrospun SiO_2_ doped Bi_2_MoO_6_ NFs degraded MB with a high photocatalytic rate under sunlight compared to pure Bi_2_MoO_6_. This enhancement is because of presence of defects on the surface of SiO_2_ and at the SiO_2_–Bi_2_MoO_6_ interface [59]. In another work, electrospun BiFeO_3_ NFs were successfully used for removing of 97% RhB. The porous BiFeO_3_ membrane also exhibit ferromagnetic behavior at room temperature with coercively ~170 Oe, saturation magnetization ~4.4 emu/g and high efficient absorbent [27]. Leindeckern et al. [51] evaluated optical properties of electrospun Nb_2_O_5_ NFs and found that the optical energy gap reduced to ~3.32 eV with increase in calcination temperature. Nb_2_O_5_ NFs has been suggested as a photocatalyst because it can be easily recovered and recycled. In another work, the catalytic property of La_0.6_Sr_0.4_Co_0.2_Fe_0.8_O_3−δ_ (LSCF) NPs coated on electrospun yttria stabilized zirconia NFs was compared with the materials produced using the conventional powder method. It was found that the exchange current of cathode made from the NFs (145.06 mA·cm^−2^) is much higher than that of powders (81.82 mA·cm^−2^), this increment has been attributed to the increase of triple phase boundary by the fiber structure [80]. Electrospun (Pd/Cu) doped CeO_2_ NFs has also been evaluated for the water gas shift (WGS) catalytic reactor used in fuel cell systems, coal gas processing, and other applications [41]. More than 60 h testing of this NFs catalyst in the WGS environment (5% CO, 10% H_2_O, balance Ar) at 400 °C indicated high WGS activity.

The electrospoun Lu_2_SiO_5_ NFs have been studied for photo-luminescent properties; and the role of Ce^3+^ in the fiber on the emission efficiency was investigated [48]. These NFs have a strong emission peak located at ~403 nm corresponding to the transitions of Ce^3+^ 5d^1^→4f^1^. Findings indicated that 1% Ce has the stronger light emission. In another similar work, the photo-luminescent properties of electrospun Eu^3+^ doped Gd_2_O_3_ NFs has been studied. The main finding of that study was an increase of the luminescent intensity and fluorescence lifetimes of Eu^3+^ doped Gd_2_O_3_ NFs with increase in concentration of Eu^3+^ or increase size of NFs [138]. Furthermore, study on the photocatalytic properties of Mo doped BiVO_4_ electrospun NFs demonstrates that doping Mo into BiVO_4_ enhance the photocatalytic activity and dark adsorption ability. Liu et al. [139] explained that a small amount of Mo doping into BiVO_4_ can efficiently separate the photo-generated carries and improve the electronic conductivity.

### 5.2. Filtration and Separation

It is necessary to implement remediation techniques to remove the organic and inorganic pollutants from gas and liquid phase polluted environment because they are harmful for ecosystems and human health. The remarkable properties of electrospun membranes, i.e., surface area, high open porosity, and interconnected porous structure, mean that they are one of the utmost promising and versatile filter media for fine particle filtration and separation. Electrospun filter media based on different polymer systems such as PA6, PA66, PAN, PU, PVA, PEO, PC, silk, copolymers such as PAN/PMMA, PVC/PU, or strengthened by ceramic such as PA-6/boehmite and PAN/TiO_2_ are widely used in filtration media [140]. 

In accordance with certain types of pollutions, appropriate single phase or composite ceramic electrospun NFs are used to remove organic and inorganic pollutants; and kinetics models have been developed for each type of pollutions. For example, Kim et al. [22] used electrospun *γ* and *β* phase of alumina NFs for adsorption of N_2_ gas and methyl orange form liquid. The isotherms of N_2_ gas adsorption by alumina NFs are in agreement with results obtained by mesoporous structure. It is found that the pseudo second order kinetic model fits better than first order in the adsorption of methyl orange. This means that NFs compose a two-dimensional mat which behaves like a surface.

Removing heavy metals is an important topic of nano filtration field. Hota et al studied sorption of Cd^+2^ ions by electrospun PA6 and PCL membrane inclusion of ceramic boehmite NPs and found that sorption capacity of polymer/boehmite is much higher (0.2 mg/L) than polymer NFs alone (0.002 mg/L) [94]. Another study demonstrated for electrospun PCL/clay and PVA/clay that NFs are suitable for use in heavy metal removal of cadmium (Cd^+2^), chromium (Cr^+3^), copper (Cu^+2^) and lead (Pb^+2^) from water due to the high surface activity [141]. Further studies showed that the adsorptive property of fly ash and photocatalytic property of TiO_2_ can introduce different functionalities on PU mat for water purification. Kim et al. [142] studied the adsorption of heavy metals (Hg, Pb) and organic element (e.g., methylene blue) by PU decorated with fly ash and TiO_2_ NPs for water purification. They found that adsorption capacity is improved in comparison to pure PU NFs. 

Oil pollution problem has prompted a necessity to develop a cost-effective and environmentally-friendly way of oil spill cleanup. Recent studies by Jiang et al. [143] showed that electrospun magnetic composite NFs can help to remove oil pollutants from waste water. Jiang et al. indicated that the electrospun magnetic PVDF/Fe_3_O_4_ NFs can be potentially useful for the efficient removal of oil in water and recovery of sorbent material [143]. In another similar work, the adsorption of organic pollutants has been investigated by magnetically separable TiO_2_-coated SrFe_12_O_19_ NFs. For this matter, first SrFe_12_O_19_ NFs were fabricated by electrospinning procedure and post-calcination, and then TiO_2_ was coated on the fiber surface by dipping those fibers in the tetrabutyl titanate solution. Li et al. [65] explained that SrFe_12_O_19_ NFs causes an improvement in the decolorizing efficiency of MB by TiO_2_ under UV–vis irradiation. Moreover, these fibers can be recollected easily with a magnet in a photocatalytic process and they effectively avoided the secondary pollution of treated water.

### 5.3. Biomedical

By reviewing recent developments in electrospun multifunctional scaffolds, it is confirmed that the designing and fabricating the scaffolds showing multiple functions has gained preliminary importance. High open porous structure, compatible mechanical strength, biodegradability and biocompatibility of electrospun scaffolds promote them as optimal microenvironment for cell proliferation, migration, differentiation, and guidance for cellular in growth at host tissue. Moreover, electrospinning can produce nanofibrous scaffolds that are highly desirable for wound dressing, drug delivery, tissue engineering and other biomedical applications [140,144,145]. 

Our investigations show that combination of biodegradable polymers with bioactive inorganic materials is necessary for biomedical application, and single phase biomaterials have limited utilization. Electrospinning method is capable to fabricate composite ceramic/polymer NFs which is requested for tissue engineering and other biomedical applications. The fibrous scaffold of HA/biopolymer could recently develop its potential in the field of tissue engineering and bone regeneration. Although electrospun membranes are famous for high surface area, which facilitates efficient adsorption of biomedical reagents, the incorporation of ceramic non-stoichiometric HA NPs inside the PA6 causes the sorption efficiency of protein BSA molecules to be improved 5 times more than one pristine PA6 NFs. By homogenous dispersion of HA NPs inside the PA6 NFs, highly porous materials (~77%) are achieved that result in sorption of 60 mg·cm^−2^ BSA molecules. The other benefit of ceramics elements using in biomedeical purification is their ability to change surface functionality and affinity; higher positive surface electric charge causes more adsorption of negative bio-molecules [127]. Moreover, addition of HA NPs into NFs modifies hydrophobicity of electrospun NFs that adhere to more favorable human cells. For this matter, Suslu et al. studied electrospun HAp/PHBV mats, and they found that surfactants strongly activate the precipitation rate of the apatite-like particles and decrease the percentage crystallinity of the HAp/PHBV mats [89]. Li et al. studied the dispersion of HA in PCL NFs and found that the tensile strength and Young’s modulus increased. Furthermore, composite NFs were examined for bioactivity and toxic in vitro tests. Findings showed that new HA formed on the HA sites and composite NFs were non-toxic to fibroblasts and osteoblasts [146]. Combination of biomimetic nanofibrous scaffolds with bio-functionalized elements is a new strategy for promoting bone regeneration, especially in bone tissue engineering. Zhang et al. [147] fabricated a zein (a kind of protein) fibrous membrane incorporated with bone matrix-mimic ceramic HA NPs by electrospinning method. It is observed that the zein/HA membranes can support cell proliferation and shows promise in bone tissue engineering applications. Su et al. [148] studied the secretion levels of Collagen I and fibronectin on electrospun PLA NFs coated by calcium silicate. They found that using inorganic apatite coatings tend to make suitable conditions for bone tissue engineering. In another work, Liu et al. [149] investigated the effect of HA/chitosan seeded with bone marrow mesenchymal stem cells (BMSC) for bone regeneration. Their findings showed HA/chitosan/BMSC is useful for bone reconstruction and tissue engineering due to the activating of intergrin-BMP/Smad signaling pathway of BMSCs on mentioned scaffold. In another research, electrospun TiO_2_ NFs have been used in a multilayer system of TiO_2_ nanofiber/graphite oxide paste/glassy carbon electrode to voltammetric determination of levodopa (l-DOPA) in aqueous media [68]. The mentioned that the electrode exhibited effective surface area, more reactive sites and excellent electrocatalytic activity due to assignment of TiO_2_ NFs. It can be mentioned that this method is capable of quantifying l-DOPA in human cerebrospinal fluid, blood serum and plasma. This is because of the good linear relationship with a limit of detection of 15.94 nM and good sensitivity of 0.0806 μAμM^−1^.

Drug delivery from electrospun fibers is an active area of research because electrospun materials are metastable superhydrophobic and hydrophobic materials and their rate of wetting controls drug release from the surface of material [150]. There are many articles published recently with regard to this application. Not only have meloxicam (MX) immobilized biodegradable chitosan/PVA/HA based electrospun NFs shown good biocompatibility, but were also confirmed to be non-cytotoxic and show very good proliferation of vero cells. It is suggested that this material may have effective utilization in periodontital treatments [151].

There are many efforts for utilization of ceramic NPs and NFs for biomedical applications. The unique antimicrobial properties as well as protein release mechanisms of SiO_2_ make the electrospun polymer-silicate hybrid NFs a candidate for wound dressing applications [82]. Suitable mechanical properties and positive magnesium release from PCL/MgO/Keratin NFs have been developed the composite materials with structural and material properties that will support biomedical applications and musculoskeletal tissue engineering [152]. The potential use of the electrospun PLA/Al_2_O_3_ NFs for biomedical application was investigated by Kurtycz et al. [87]. They found that the PLA/Al_2_O_3_ NFs mat is not toxic in indirect cytotoxicity evaluation with human skin fibroblasts. Furthermore, cell culture studies revealed that cells had normal shapes and are integrated well with surrounding NFs. In another research, Guo et al. [58] prepared Ag/SiOC composite NFs via electrospinning method and possessed antibacterial activity for both Gram-negative *E. coli* bacteria and Gram positive s. aureus bacteria. It was explained that Ag/SiOC composite NFs are a promising material for antibacterial filtration application. Eventually, electrospun PCL/CaO NFs containing biodegradable and ceramic particles are used for tissue engineering [153]. Antibacterial activity results of the above-mentioned NFs show non activity, and MC3T3-E1 cell viability demonstrate the highest levels of activity for CaO-loaded matrices containing gelatin after 7 days in culture. Therefore, CaO NPs loaded electrospun mats could be a potential material for application in bone tissue engineering.

### 5.4. Fuel Cells

Many recent researches have focused on new materials for intermediate temperature solid oxide fuel cells (SOFSc) due to long term stability of electrochemical activity and low energy consumption [55]. Catalytic activities of the cathode materials in SOFCs depend on oxygen ionic conductivity and oxygen transport kinetics. Besides, the cathode performance is also closely related to the microstructures such as porosity, particle sizes and particle connectivity. Hence, Liu et al. [55] synthesized Pr_0.4_Sr_0.6_Co_0.2_Fe_0.7_Nb_0.1_O_3−δ_ (PSCFN) NFs via electrospinning method to evaluate their usefulness as cathode in SOFCs. The PSCFN NFs was infiltrated by Gd_0.2_Ce_0.8_O_1.9_ (GDC) precursor to provide the composite cathode. It was found that the PSCFN–GDC (1:0.10) had excellent stability of electrochemical activity under a current density of 200 mA·cm^−2^ for 100 h at 800 °C. Their findings prove that the PSCFN–GDC composite NFs can act as a highly efficient cathode candidate for the intermediate temperature SOFCs. In another work, electrospun GdBaCo_2_O_5+δ_ (GBCO) NFs calcined at 1000 °C were used as a cathode for electrochemical performance analyses. It is suggested that this procedure is time- and cost-saving and easy for manipulation as compared with the fabrication process using sol-gel method. Furthermore, the homogenous network structure of the GBCO cathode prepared with electrospinning route is believed to enhance the cathode electrochemical activities and realize improved performance. It is also suggested that it can serve as a promising cathode material for intermediate temperature SOFC [38]. The performance of electrospun carbon NFs supported Pt catalyst as electrodes and hydrocarbon based sulfonated polyether ether ketone (SPEEK) as electrolyte in proton exchange membrane fuel cells have been investigated by Padmavathi et al. [154]. They show that, compared to commercially available Pt/C catalyst and Nafion-117 membrane, the electrospun carbon /Pt NFs membrane showed higher power density (294.7 mW/cm^2^).

### 5.5. Sensors

By reviewing the recent developments of electrospun ceramic NFs, it is recognized that these materials can construct a powerful platform to understand and design practical sensors. This is because of their high open porous structure and good mechanical strength. On the other hand, loading nano functionalized elements on the electrospun scaffolds promote sensing ability of these materials in biomedicine, waste water and gas treatment, air filtration and other utilizations.

There are many studies about the potential application of electrospun NFs with regard to biosensors. Stafiniak et al. evaluated the electrospun ZnO NFs biosensor by a novel method based on the standard microelectronic device technology and using AlN_x_ amorphous thin film [72]. They found that the reversible response to physiologically relevant BSA concentrations in aqueous solution reached a high sensor current. In another work, the electrochemical response of LaMnO_3_ fibers modified carbon paste electrode (LaMnO_3_/CPE) for fructose determination has been evaluated in the 0.4–100 μM range and a low detection limit (0.063 μM) was found in comparison with other modified electrodes [44].

Semiconducting SnO_2_, ZnO, TiO_2_, and CeO_2_ NFs are widely used as gas sensors due to changes in the number of electric charge carriers caused by reduction/oxidation reactions occurring at their surfaces [62]. Therefore, attention to the surface area and surface activity are the key factors for ceramic NFs in gas sensing application. Recent research demonstrates the potential of ultra- sensitive gas detection at low operating temperature. Kim et al. [60] used SnO_2_ NFs as a gas sensing device for NO_2_ and CO gases by new synthesis method. In comparison to conventional micro scaled gas sensor devices, prototypes comprising of a random network of electrospun SnO_2_ NFs do not require higher operation temperatures. The detection limit of SnO_2_ NFs gas sensor device is 150 ppb NO_2_ at 185 °C. 

Furthermore, the chemical composition of NFs has a significant role in sensing of elements. Xu et al. found that not only is hydrogen sensing performance improved by doping Al into SnO_2_ NFs, but also response time (∼3 s) and recovery (less than 2 s) become rapid. It is believed that changing the crystals of SnO_2_ NFs by incorporation of Al is main reason for this phenomenon [61]. Similar results have been observed by doping the Eu^3+^ cations into SnO_2_ enhancing significantly sensing ability of pure SnO_2_ NFs [63]. 2 mol % Eu doping to SnO_2_ causes increasing sensing two times higher than that of the pure SnO_2_ NF sensor at an operating temperature of 280 °C. In another work, SnO_2_–CeO_2_ composite NFs exhibited the highest response to ethanol. This is because catalytic activity of CeO_2_ is not formed in compositions of Ce content lower than 6 mol % [62].

It is worth mentioning that, with the modification of the microstructure and fabrication process, high sensitive NFs can be achieved. Samanta et al. [155] explained that parallel electrospun ZnO NFs can be used for detection of lower concentration gasses (lower than 15 ppm) due to crystal structure and orientation of ZnO NFs. Among the different kinds of ZnO microstructure for sensing gases (e.g., acetone), Wei et al. [156] investigated the bristlegrass-like ZnO NFs for acetone sensing. They showed that electrospun products have fast response, good selectivity and repeatability in acetone sensing at 215 °C, which it is attributed to the bristlegrass nanostructure. In another work, Giancaterini et al. [69] reported a relative response of ~12.4 and 97% of full recovery using electrospun WO_3_ NFs which enabled NO_2_ sensing as low as 400 ppb.

Another sensing aspect of electrospun NFs is to detect heavy metals, nitrate, carbonate and other elements in waste water or air that make online monitoring of pollutants in real environments. Electrospun G/PANI/PS hybrid NFs have been used in an electrochemical sensor to sense the Pb^2+^ and Cd^2+^ due to the high surface area and electrical conductivity [84]. In this case, a linear range of 10–500 μg·L^−1^ was obtained for both Pb^2+^ and Cd^2+^; and limits of detection were found to be 3.30 and 4.43 μg·L^−1^ for Pb^2+^ and Cd^2+^, respectively. Hollow ZnO NFs have been investigated as an explosive nitro-compounds sensor and it was found that these NFs could successfully sense the nitro compounds; however, the sensing performance is greatly affected by the molecular structure of the nitro compounds [73]. In another work, Pascariu et al. [157] suggest that NiO–SnO_2_ NFs can be used as active nanostructures for humidity sensors due to the electrical results obtained under humidity. They believe that the significant effective surface of NiO–SnO_2_ NFs is the main reason for increasing the conduction in the water environment. Furthermore, the porous electrospun Li^+^ doped SnO_2_ NFs also exhibited ultrafast response and recovery time within 1 s at a relative humidity level of 85%. Hence, the electrospinning method provides ultrafast sensors for practical applications, especially fast breathing sensors [158]. By using the one dimensional electrospun core-shell TiO_2_-Al_2_O_3_ NFs online sensing to H_2_S, CH_3_OH and C_2_H_5_OH in N_2_ background is possible, but it should be mentioned that sensibility is not the same for all pollutants and the highest amount (three times more than the others) has been recorded for C_2_H_5_OH gas [159].

Application of electrospun NFs are not included only to above items; however, new application can be assumed for these materials. Zheng et al. [90] could successfully synthesize twisted PVDF/CNT composite NFs via modified electrospinning procedure. In comparison, with aligned arrays, twisted PVDF/CNT composite fiber ropes showed enhancement in mechanical and electrical properties. By adding more CNT into PVDF NFs (16.7%) tensile strength improved 3.5 times and electric resistance decreased from about 6 to 2 MΩ. Therefore, microscale strain sensors application for electrospun PVDF/CNT composite products is assumed. We know that bulk ceramic based sensors are usually used for high temperature sensor applications. However, negative temperature coefficient like NiO can be used as thermally sensitive resistor element in low temperature range. The temperature sensor performance of the electrospun NiO NFs has been examined by George et al. [53] in 30–100 °C temperature range. A linear trend for electrospun NiO NFs was observed that makes this material suitable for thermistor applications.

### 5.6. Batteries

Lithium ion batteries (LIBs) have attracted increasing attention due to their high energy density, long cycle life, lightweight and low environmental impact. Recent efforts have been focused on finding new electrode materials including new composition and attractive microstructures similar to the electrospun NFs. For example, mesoporous CNT/electrospun carbon NFs electrodes are applied as a binder-free electrochemical electrode for the LIB. The super high porosity mat presents many adsorption sites of lithium ions, and higher electrical conductivity [160]. The electrospun carbon NFs interlayers induce the Li ions to form uniform Li metal deposits on the fiber surface and in the bulk to strengthen the cycling stability of the Li metal anodes [161]. In the following, activities on electrospun SiO_2_, Al_2_O_3_ and SnO_2_ based NFs for LIB are reviewed.

Electrospun silica (SiO_2_) fibers with average diameter of ~700 nm are added to a ternary poly (ethylene carbonate)-lithium bis (trifluoromethanesulfonyl) imide-ionic liquid solution for use in LIBs [162]. It is showed that the mechanical stability and freestanding of composite membranes are improved by the reinforcing effect of silica NFs homogeneously into polymer matrix. Furthermore, conductivity of 10^−5^ S·cm^−1^ at 80 °C and favorable Li transference number of 0.36 are other achievements of using electrospun SiO_2_ NFs in LIB application. In another work, which assigned SiO_2_ NPs inside the electrospun P(VdF-HFP) NFs [163], it is concluded that in-situ incorporation of SiO_2_ NPs improves the electrical properties more than that achieved by directly mixing of silica to the polymer. Maximum ionic conductivity of 8.06 mS·cm^−1^ at 20 °C was achieved with 6% in situ silica. Appropriate electrolyte uptake (>550%) by high porosity (∼90%) electrospun membrane is another advantage of these materials in LIBs. 

Electrospun hybrid P(VdF-co-CTFE) and Al_2_O_3_ composite membrane made by Lee et al. [86] has been used in LIBs. It is found that thermal stability and cycling performance enhanced due to effective encapsulation of the electrolyte solution into good microporous structure of electrospun membranes. In another work to explore the effect of ceramic composite separators on the thermal shrinkage and electrochemical performance of the separators in LIBs, a nano sized Al_2_O_3_ coating was applied on both sides of microporous polyethylene (PE) separator [164]. It is worth mentioning that the immiscible coating solution presents superior electrochemical performance, whereas the miscible coating solution shows the better thermal shrinkage. Furthermore, the microporous structure of ceramic coating affects the thermal shrinkage as well as the electrochemical performance of ceramic composite separators.

The electrochemical performance of the electrospun ZnO/SnO_2_ composites for use as anode materials in LIBs has been investigated by Luo et al. [75] with regard to the effect of heat treatment on the efficiency of charge and discharge capacities. They found that calcination at 700 °C not only delivered high initial discharge and charge capacities of 1450 and 1101 mAh·g^−1^, respectively, with a 75.9% coulombic efficiency, but also maintained a high reversible capacity of 560 mAh·g^−1^ at a current density of 0.1 Ag^−1^ after100 cycles. In the other work that suggests improvements in the chemical properties of Ge-based anode materials, composite GeO_2_/SnO_2_ NFs were investigated for LIB application. It is found that GeO_2_ concentration has impact on enhancement of cycle stability of NFs as an anode. At the optimized concentration (Ge/Sn: 0.88), high initial reversible capacity of 922 mAh·g^−1^ and excellent cyclability (charge capacity retentions ~73.9%) were achieved [39].

The room temperature ionic electrolyte made by electrospinning method is an alternative for the replacement of organic electrolytes. Raghavan et al. [91] examined nano-sized ceramic fillers (SiO_2_, Al_2_O_3_ or BaTiO_3_) hosted in electrospun P(VdFHFP) membranes for use in high energy density LIBs as a polymeric electrolyte. It is observed that composite ceramic NPs/Polymer NFs have good interfacial stability and oxidation stability at 5.5 V, and it is elucidated that the highest achievable potential of 6 V is belonged to membrane including BaTiO_3_ NPs. Furthermore, in comparison to the other membranes, this membrane delivered high initial discharge capacity of 165.8 mAh·g^−1^, which corresponds to 97.5% utilization of active material under the test conditions and showed the capacity fade after prolonged cycling. In another work, the incorporation of ceramic fillers (SiO_2_ and TiO_2_) inside a thermoplastic polyurethane (TPU)/PDdF based gel polymer electrolytes for LIB was studied. Based on the high ion conductivity (4.8 × 10^−3^ S·cm^−1^) and mechanical performance (8.7 ± 0.3 MPa) at room temperature, Wu et al. [165] suggest that TiO_2_ is more efficient in improving the properties of gel polymer electrolyte for practical application. In another research, Shim et al. [42] introduced electrospun LaCoO_3_ NFs for oxygen reduction and evolution in rechargeable Zn–air batteries. They explained that the LaCoO_3_ NFs have better electrochemical properties compared with the LaCoO_3_ powder, which is attributed to the increased surface area and number of active sites in the fibers.

### 5.7. Electronic Devices

Electrospun NFs have the potential to be used in many electronic devices due to their high surface area, open porous microstructure and multi-composition. Hence, there are many efforts to discover new advanced materials fabricated via electrospinning method based on the electric and magnetic properties. Schutz et al. [37] could successfully create Cu_2_ZnSnS_4_ (CZTS) phase via electrospinning procedure and post heat treatment. The NFs characterization confirms the microstructure, composition and morphology of a homogeneous compact film, as is required for the production of photovoltaic cells. In other research, Ghashghaie et al. found that the electrospun ZnO NFs are capable to assemble into the inter-electrode space via dielectrophoresis force in above of 1 kHz (5 and 20 kHz) frequencies. Therefore, it is observed that ZnO NFs are aligned along the electric field lines thereby indicating desirable conditions for electronic device applications [21].

### 5.8. Supercapacitors and Energy Harvesting Systems

The development of renewable and sustainable energy sources is one of the main topics of recent researches due to decline of natural resources. Among the energy storage systems, supercapacitors and energy harvesting systems have specific attentions. Carbon-based NFs have been considered promising electrodes for advanced electrical energy storage systems, e.g., rechargeable batteries and supercapacitors, because of their high conductivity, good mechanical integrity, and large surface area [166]. Hence, Wang et al. [29] applied electrospun CNFs substrates coated with a uniform ceramic MnO_2_ ultrathin layer by dip coating method for using in electrochemical capacitors. Based on the characteristics obtained for composite electrode (specific capacitance ~557 F·g^−1^), good rate capability and long-term cycling stability were observed. It is suggested that CNFs/MnO_2_ nanocomposites are promising for high-performance supercapacitors. In contrast, in another research, it was found that the best energy harvesting performance is obtained for pure PVDF NFs, with power outputs up to 0.02 µW and 25 µW under low and high mechanical deformation. Composite making with BaTiO_3_ NPs results in reduction of power output. It is because of enhancement of mechanical stiffness. It is suggested that the power output of the composites being better for the nonpiezoelectic smaller fillers [93]. However, in accordance with the results obtained by Baji et al. [167], piezoelectric hysteresis and ferroelectric switching behaviors of electrospun (BaTiO_3_)/(PVDF) composite NFs are recognized. They investigated ferroelectric properties of the above-mentioned NFs by using piezoresponse force microscopy and found the polarization-voltage and amplitude-voltage hysteresis loops for (BaTiO_3_)/(PVDF) NFs.

### 5.9. Magnetic Parts

Electrospinning procedure enables the facility to obtain nanocrystalline materials that have particularly important effects on magnetic materials. Recently, optimistic findings have been published throughout the electrospun magnetic NFs. Yensano et al. [45] studied the magnetic properties of electrospun La_0.7_Sr_0.3_MnO_3_ and they found that the specific saturation magnetization (Ms) value of calcined NFs at 900 °C is 40.52 emu·g^−1^ at 10 kOe. The increase of Ms is consistent with the enhancement of crystallinity and crystallite size by considering a magnetic domain of the samples. In another work, the magnetic properties of Ce_0.96_Fe_0.04_O_2_ NFs were investigated by S. Sonsupap et al. [33]. It is found that as-spun samples (PVP/Ce_0.96_Fe_0.04_O_2_) exhibit a diamagnetic behavior, whereas the calcined Ce_0.96_Fe_0.04_O_2_ samples at 500–800 °C is ferromagnetic with the specific magnetizations of 0.002–0.923 emu·g^−1^ at 10 kOe. Hence, it is suggested that the electrospun Ce_0.96_Fe_0.04_O_2_ NFs can be further developed for many applications including ferrofluids, magnetic recording, biomedicine, and spintronics. Furthermore, Liu et al. [25] prepared BaFe_12_O_19_ fibers and hollow fibers by electrospinning and coaxial electrospinning method, respectively. They elucidated that the hollow NFs had low coercivity values of a few hundred Oersted while NFs have more than a thousand Oersted. They also found that the hollow NFs exhibited strong magnetism and basically showed soft specification. It is suggested that BaFe_12_O_19_ hollow NFs are promising for use in a number of applications, such as switching and sensing, electro-magnetism, and as microwave absorbers.

### 5.10. Dielectrics

Ceramics are used in many electromagnetic interference shielding applications due to their appropriate dielectric characteristic. Electrospun products have a significant role in achieving multifunctional dielectric materials. In a study, the real and imaginary permittivity of carbon NFs was increased 3.5 times by incorporation of ZrO_2_ NPs, and the best efficient electromagnetic interference shielding effect (31.79 dB in 800–8500 MHz) is achieved when the amount of ZrO_2_ NPs is increased and heat treatment is carried out at 2100 °C [88]. In other research, Qin et al. [30] found that CaCu_3_Ti_4_O_12_ NFs fabricated by standard electrospinning has a different dielectric constant from those synthesized by conventional bulk methods. It is suggested that NFs not only provide a new topic for investigation, but also supply new high-performance devices in electronic applications.

### 5.11. Thermoelectric Materials

Composite NFs fabricated via electrospinning generate advanced materials for the conversion of waste heat into electricity as thermoelectric materials, due to enhanced phonon scattering at the nano-grain boundaries. Thermoelectric figure of merit (ZT) of electrospun boron-doped barium-stabilized bismuth-cobalt oxide have been studied by Cinar et al. [28]. The physical measurement system values showed that the electrical and thermal conductivity, the Seebeck coefficient, and the ZT increased with the temperature rise. In contrast, they found that the ZT values decreased with doping of B. In other words, boron doping had a negative effect on the thermoelectric Ba-Bi-Co-O system. Thermoelectric and humidity sensing analysis of electrospun La_2_CuO_4_ NFs are also carried out by Hayat et al. [43]. Their findings in the analysis of Seebeck and the analysis of impedance of La_2_CuO_4_ NFs indicated that the Seebeck coefficient increased from ∼30 to ∼300 µV·K^−1^ at 298–308 K, and the space-charge polarizations easily followed the changing direction of the electric filed at 100 Hz. They confirmed that the narrow adsorption-desorption hysteresis, short response and recovery time, excellent repeatability, high stability and high sensitivity of La_2_CuO_4_ NFs originated from a high surface to volume ratio of electrospun NFs, which enable them to be used as a humidity sensors.

### 5.12. Conductive Wires

The advances in electrospun ceramic nanowires have brought an increasing interest in the potential technological applications such as those mentioned above as well as light-emitting diodes (LEDs), flexible displays, solar cells, organic LEDs, touch screens, and bio-textiles. Conductive fillers and metal conductive particles filled with flexible substrates can be easily formed by electrospinning method, such as silver NWs/PET, indium tin oxide (ITO)/PES, MWCNT and single wall carbon nanotubes (SWCNTs) [168]. 

Conductive electrospun products show promising applications in various tissue engineering because of their higher conductivity. The neural tissue engineering is improved with the PPy, PANi and PEDOT fibrous conductive scaffolds. Also, conductive materials such as PPy, PANi, PLGA/CNF, CNTs and CNTs coated electrospun products are successfully utilized as scaffold materials for cardiac tissue engineering [169,170]. Gaminian et al. [171] fabricated cellulose NFs decorated with Ag NPs by electrospinning followed by the deacetylation method. They believed that multi-functional cellulose NFs that are achieved by this method would provide biodegradable materials for various applications with a minimal amount of potentially toxic materials. Furthermore, not only is the electrical resistivity of cellulose NFs decorated with Ag NPs low (around 35 KΩ per square) but also their tensile strength is 87% higher than pristine cellulose NFs.

There are a lot of reports on semiconductor NWs exploring excellent sensing properties due to high surface area. However, the sensing ability can be promoted by modification of morphologies. Liu et al. [172] explored that acetone sensitivity based on In_2_O_3_ nanotubes (NTs) is better than corresponding solid NWs. They suggest that the one-dimensional NT is probably a better candidate than NW for the higher response in the actual applications. On the other hand, pure and single phase ceramic NWs have also different performance than ceramic/polymer NWs. Chiu et al. [173] synthesized CuCrO_2_ NWs by electrospinning method. They found that the calcination conditions play a significant role in achieving single phase CuCrO_2_ NWs for use in p-type transparent optoelectronic devices. 

### 5.13. Wearable and Electronic Textiles 

Nowadays, rapid developments in nanotechnology create a new application of electronic devices that are miniaturized to the point where embedded wearable applications are beginning to emerge [174]. Electrospinning is one of the methods for production of wearable, smart and electronic textiles with multi functionality, flexibility, conductivity, low energy consumption, and miniaturization [175]. Wearable mats are electronic and smart textiles that represent a useful feature for power management, and many electronic devices, fabrics, and bio-tissues will have to meet special requirements concerning wearable textiles [176]. It is worth mentioning that wearable textiles are an innovate approach for converting mechanical movements to electrical power, and undoubtedly they will come to the market based on the recent successful results obtained by pioneer researchers. Hu et al. [177] successfully immobilized Ag NPs into electrospun Na-alginate NFs via a novel, cost-effective and antibacterial approach for using as flexible electronic skin. They explained that stable response of Ag/alginate nanofibrous membrane is because of uniform distribution of Ag NPs inside the alginate NFs. The electrospinning method provides conditions for them to synthesize the practical wearable electronic textiles that have an ultralow detection limit of 1 Pa and high durability more than 1000 cycles. Therefore, they suggest that electrospun Ag/alginate can be used as a pressure sensor on uneven human skin to sense respiration and vocal cords vibrations.

A recent interest in the utilization of electrospun wearable electronic NFs is transparent human hair-based textiles. This is because of their unique optical properties in the visible light region. In order to apply wearable electronic devices with transparent textiles, Lee et al. [178] fabricated transparent ZnO/graphene quantum dots textiles via electrospinning method and found that the luminescence of these textile LEDs devices is ~70.19 cd·m^−2^. Park et al. [179] successfully synthesized environmentally friendly human hair-based, transparent, keratin/PVA NFs via electrospinning method. They investigated a comparison between polymer light-emitting diodes (PLEDs) without textile and consolidated PLEDs with textile for study the transparency of NFs for wearable devices. The performance with a spectrally white, red and yellow color light of consolidated textile/PLEDs/textile devices indicated a maximum luminance of 2791, 2430, and 6305 cd·m^−2^, and a current efficacy of 0.29, 0.10, and 0.38 cd·A^−1^, respectively. Their findings indicate that consolidated wearable devices with the PLEDs embedded in the environmentally friendly transparent NF textiles opened a new world of applications for wearable electronics.

Energy storage materials have significant roles in energizing portable and wearable electronics. Assessment of multi hierarchical constructions those fabricated via the electrospinning procedure enhance the ability of flexible supercapacitor electrodes. Activated carbon fibers such as flexible substrates, PANI and CNT as conductive materials can make a high performance mats for flexible textile electrodes that have good cycling stability, energy density and power density [180]. Furthermore, piezoelectric materials respond to wearable smart textiles because they can convert mechanical energy into electrical energy through a piezoelectric effect. The piezoelectric properties of PVDF NFs embedded BaTiO_3_ NPs have been evaluated toward NPs concentration by Lee et al. [92]. Their findings showed that the magnitude of the resultant voltage increases as the NPs concentration increases. The piezoelectric output voltages of PVDF/BaTiO_3_ were 1.7 times greater than single phase PVDF NFs. Moreover, uniaxially-aligned PVDF/BaTiO_3_ NFs suggest possible uses in energy harvesting and as power sources in miniaturized electronic devices like wearable smart textiles and implantable biosensors.

The next generation wearable textiles will belong to nanofibrous membranes that are capable of converting human biomechanical energy into electricity. Some efforts are under development to construct bio electric nanogenerators. This is of vital importance to portable energy-harvesting and personal electronics. Electrospinning provides portable, and wearable self-powered nano/microsystems that require the piezoelectric materials to be flexible and lightweight. Li et al. [181] have demonstrated that the electrospun nanofibrous membranes are tailored to enhance the polarity, mechanical strength as well as surface hydrophobicity of bio electric nanogenerators, which will eventually improve the device performance, power, and capability of operation even with high environmental humidity. Wu et al. [175] synthesized a textile with parallel NWs of lead zirconate titanate (PZT) by electrospinning method for using it into flexible and wearable nanogenerators. The electrospun PZT NWs can generate an output voltage of ~6 V and output current of ~45 nA, which are large enough to power a liquid crystal display and a UV sensor, as well as powering wearable microsystems [174]. 

### 5.14. Other Applications

Similar to the common fibers which are applied for reinforcing the bulk materials, electrospun NFs are also used to increase mechanical strength. Calleja et al. [26] prepared a thin film of YBa_2_Cu_3_O_7__−__x_ embedded electrospun fiber network of BaZrO_3_ and investigated this for mechanical strength. They found that mechanical performance of the composite enhanced due to the presence of NFs. Not only a mechanical reinforcement, but also other interesting composite materials can be designed based on the using electrospun NFs. Electrospun SiO_2_ NFs have been examined as coating to the ceramic tile surfaces. It is worth mentioning that by electrospinning technique, microscopic defects of tile surface can be covered with NFs [56]. In a different application of electrospun ceramic NFs, hybrid configuration of nafion/silica NFs were examined for fire resistance properties and wettability. It is found that not only were the thermal properties of nafion enhanced by chemical bonding with silica NFs, but also fire resistance improved with porosity features, which could effectively prevent fire speed and heating flow. In organic–inorganic sols, the phase rearrangement is induced by applied high voltage field, which leads to highly conductive polymer being forced to the surface of composite fiber to form shell to protect the inner inorganic materials [182]. 

According to the sound absorption properties of electrospun mats, Gao et al. [183] assigned electrospun PVA/TiO_2_ and PVA/ZrC composite mats for using a spiral vane electrospun machine. They carried out the sound test in the impedance tube at the frequency range from 500 to 6500 Hz. It is found that sound absorption properties of composite shifted to a higher frequency region when ZrC NPs loaded, and better sound absorption properties seen above 2500 Hz with increasing content of ZrC. For TiO_2_ NPs, the size of NPs is the main variable in terms of adsorbing sound. Therefore, it can be said that the NPs had an effect on sound absorption materials, with different types and sizes, and sound absorption properties will improve in different ranges of frequency.

The reducibility of the electrospun Ce_x_Sm_1−x_O_2_ NFs as well as their thermal stability in successive oxidation–reduction cycles has been evaluated in H_2_ atmosphere by Jaoude et al. [34]. They found that the Ce_x_Sm_1−x_O_2_ NFs have mobile oxygen species (reducible sites) and a wide range of acid/basic sites. Furthermore, the Ce_x_Sm_1−x_O_2_ NFs enhanced the reactive adsorption of ammonia leading to the production of NH_3_, NO, N_2_O and N_2_O species. According to the obtained results, they suggest Ce_x_Sm_1−x_O_2_ NFs for use in energy-related industrial applications such as hydrocarbons steam reforming, water gas shift reaction and cracking reactions.

## 6. Summary and Future Perspectives

Ceramic NFs can be synthesized via several methods. However, the electrospinning method has significant advantages over the others because it is straightforward, cost-effective, and versatile and can produce ceramic fibers in the nanometers to micrometers range. Utilization of ceramic NFs instead of bulk ceramics improves the performance of devices due to special properties that come from electrospinning products. Electrospinning is a practical method to produce ceramic fibers in a variety of shapes: one dimensional, tubular, hollow, core-shell, and porous. Not only the shapes of fibers, but also tha pattern of fibrous mats can be changed via electrospinning procedure: non-woven, cross and aligned fibers, 3D mats and ropes. 

Single phase ceramic fibers are synthesized by calcination of electrospun hybrid ceramic/polymer fibers. Polymer has significant role to obtain appropriate viscosity for pre-spinning solution. PVA and PVP are fairly common polymer reagents for the above-mentioned purpose. By reviewing recent developments in electrospun ceramic NFs, it is found that not only can simple oxide ceramic fibers such as, Al_2_O_3_, MgO, SiO_2_, TiO_2_, ZnO and ZrO_2_ be fabricated via electrospinning method, but also complex oxide ceramic fibers such as CaCu_3_Ti_4_O_12_ and Li_1.6_Al_0.6_MnO_4_ can be easily synthesized. The surface activity of ceramic NFs can be improved by post treatment like pyrolysis, hydrothermal and carbothermal processes. The integration of electrospinning with surface modification procedure presents a pioneering method for fabrication of complex non-oxide ceramic NFs (e.g., Cu_2_ZnSnS_4_), high crystallized fibers, and they are never synthesized via other methods. 

With the decrease in diameter and length of a fiber, many properties of fibrous materials are modified, and characterization of NFs seems to be different from bulk materials. For example, the zeta potential of ceramic NFs are measured by the different procedure and setup compared to the one for powders and common materials. 

The recent findings have shown the great potential of electrospun ceramic NFs to be used for making various catalyst parts, filtration media, sensors, electronic devices, magnetic parts, wearable electric textiles, and biomedical ones. We can summarize recent ceramics NFs synthesized via electrospinning in following list according to their applications.

■ ***Catalysts***: TiO_2_, V_2_O_5_, ZnO, SnO_2,_ CdTiO_3_, Bi_2_MoO_6_, Nb_2_O_5_, Gd_2_O_3_
■ ***Filtration***: TiO_2,_ Al_2_O_3_, Clay, Fe_3_O_4_, SrFe_12_O_19_
■ ***Biomedical***: HA, CaO, SiOC, TiO_2_, ZnO
■ ***Fuel Cells***: Pr_0.4_Sr_0.6_Co_0.2_Fe_0.7_Nb_0.1_O_3−δ_, GdBaCo_2_O_5+δ_
■ ***Sensors***: SnO_2_, ZnO, TiO_2_, CeO_2_, NiO, LaMnO_3_
■ ***Batteries***: SiO_2_, Al_2_O_3_ SnO_2_, GeO_2_, BaTiO_3_, LaCoO_3_
■ ***Electromagnetic devices***: Cu_2_ZnSnS_4_, ZnO, BaO, La_0.7_Sr_0.3_MnO_3_, Ce_0.96_Fe_0.04_O_2_, BaFe_12_O_19_, CaCu_3_Ti_4_O_12_, ZrO_2_, La_2_CuO_4_
■ ***Energy harvesting and capacitors***: BaTiO_3_, MnO_2_, In_2_O_3_
■ ***Wearable electric textiles***: ZnO, Geraphene, CNT, BaTiO_3_, PZT
■ ***Other applications***: Al_2_O_3_, BaZrO_3_, SiO_2_, ZrC, Ce_x_Sm_1−x_O_2_

It should be noted that above list does not include all progress around the world: there are definitely now many efforts being studied in research laboratories, and with further progress in electrospinning techniques, electrospun ceramic NFs will come into the market and be utilized in many devices in the not-too-distant future.

## Figures and Tables

**Table 1:** Recent developments in single phase electrospun ceramic NFs.

Ceramic Fiber	Ceramic Precursor(s)	Polymer Reagent(s)	Calcination Condition(s)	Morphology of Fiber	Application	Ref.
Al_2_O_3_	aluminum isopropoxide	PVP	500–1100 °C	Straight	Surface adsorption	[22]
Al_2_O_3_	Al_2_Cl(OH)_5_·2.5H_2_O,	PVA	1100 °C–1 h	Straight	Reinforcement	[23]
Al_2_O_3_ with CaO–SiO_2_	AlCl_3_·6H_2_O, Ca(NO_3_)_2_·4H_2_O, Si(OC_2_H_5_)_4_	PVP	600, 800, 1300 °C–1 h	Straight	Insulation area	[24]
BaFe_12_O_19_	Ba(NO_3_)_2_, Fe(NO_3_)_3_·9H_2_O	PVP	800 °C–2 h	Hollow fiber	Switching and sensing applications, Electro-magnetic materials, microwave absorber	[25]
BaZrO_3_	Barium acetate, zirconium 2,4-pentadionate	PVP	800 °C–2 h	Straight	Superconductor magnets, motors and generators	[26]
BiFeO_3_	Bi(NO_3_)_3_·5H_2_O, Fe(NO_3_)_3_·H_2_O	PVP	350 °C–0.5 h (Argon atmosphere)	Composed of NPs together	Photocatalytic activity	[27]
Ba-stabilized Bi-Co oxide	cobalt (II) acetate, barium acetate, bismuth (III) acetate	PVA	850 °C–2 h	Straight	Thermoelectric application	[28]
MnO_2_	KMnO_4_	PAN	1000 °C–2 h	Diversified texture	Electrochemical Capacitors	[29]
CaCu_3_Ti_4_O_12_	Cupric acetate, calcium nitrate, tetrabutyl Titanate, 2,2-bis(4-cyanatophenyl) isopropylidene	PVP	600–1130 °C	Straight with beads	Dielectric	[30]
CaCu_3_Ti_4_O_12_	Ti(C_4_H_9_O)_4_, Cu(NO_3_)_2_·3H_2_O, CuCl_2_, Ca(NO_3_)_2_·4H_2_O, CaCl_2_	PVP	900 °C–4 h	Straight	Fillers in dielectric	[31]
CdTiO_3_	Cd(CH_3_COO)_2_·2H_2_O, TIP	PVA	800 °C	Smooth and uniform surface	Removal of industrial pollutants and noxious wastes	[32]
Ce_0.96_Fe_0.04_O_2_	Ce(NO_3_)_3_·6H_2_O, Fe(NO_3_)_3_·9H_2_O	PVP	500, 600, 700, and 800 °C for 2 h	Straight	Magnetic applications	[33]
Ce_x_Sm_1−x_O_2_	Ce(NO_3_)_3_·6H_2_O, Sm(NO_3_)_3_·6H_2_O	PVP	500 °C–2 h	Short fiber	Energy industrial applications	[34]
CoFe_2_O_4_	Co(NO_3_)_2_·6H_2_O, Fe(NO_3_)_3_·9H_2_O	PVA	300, 500 and 800·°C for 4 h	Straight	Magnetic recording device	[35]
CuCr_2_O_4_	Cupric nitrate and Chromium acetate	PVP	500–800 °C–2 h	Particles sintered after heat treatment	Catalysts	[36]
Cu_2_ZnSnS_4_	Cu(CH_3_COO)_2_, Zn(CH_3_COO)_2_, SnCl_2_, thiourea	PVB	150–550 °C, 1–48 h	Sintered after heat treatment, Laminated, Sintered particles	Photovoltaic cell	[37]
GdBaCo_2_O_5+δ_	Gd(NO_3_)_3_·6H_2_O, Ba(NO_3_)_2_, Co(NO_3_)_2_·6H_2_O	PVP	600, 900 and 1000 °C for 5 h	Sintered particles	Solid oxide fuel cell	[38]
GeO_2_/SnO_2_	Tin(II) chloride, germanium oxid	PVP	500 °C–2 h	Straight	Lithium-ion batteries	[39]
HA	Ca(NO_3_)_2_·4H_2_O, P_2_O_5_	PVP	500–700 °C–0.5 h	Straight	Biomedical	[40]
Pd/Cu doped in CeO_2_	Ce(NO_3_)_3_·6H_2_O, Pd(NO_3_)_2_·2H_2_O, Cu(NO_3_)_2_·2H_2_O	PVP	550 °C	Straight and smooth	Water-Gas Shift (WGS) catalysis	[41]
LaCoO_3_	La(NO_3_)_3_ 6H_2_O, Co(NO_3_)_2_·6H_2_O	PVP	200, 400, and 700 °C–2 h	Short fiber	Rechargeable Zn–air batteries	[42]
La_2_CuO_4_	La(NO_3_)_3_·6H_2_O, Cu(NO_3_)_2_·2.5H_2_O	PVP	600 °C for 5 h	Straight	Humidity sensor	[43]
LaMnO_3_	La(NO_3_)_3_·6H_2_O, Mn(Ac)_2_·4H_2_O	PVP	600 °C–3 h	Bend fibers after heat treatment	Sensors	[44]
La_0.7_Sr_0.3_MnO_3_	LaN_3_O_9_·6H_2_O, Sr (NO_3_)_2_, Mn(NO_3_)_2_·4H_2_O	PVP	500, 700, and 900 °C for 7 h	Continuous structures, packed particles	Magnetic properties	[45]
La_2_Zr_2_O_7_	Basic zirconium carbonate, La(NO_3_)_3_·6H_2_O, LaCl_3_·6H_2_O, La(CH_3_COO)_3_·4H_2_O	PVA	600 °C–2 h	Sintered particles to form a fiber	High temperature insulation applications	[46]
Li_1.6_Al_0.6_MnO_4_ doped Al	Lithium acetate, manganese nitrate and aluminum nitrate	PVA and PVP	500,700,900 °C–2 h	Short and Straight fiber, relatively parallel	Lithium adsorption from polluted effluents	[47]
Ce doped Lu_2_SiO_5_	Lu(NO_3_)_3_, Ce(NO_3_)_3_, Si(OC_2_H_5_)_4_,	PVB	1000–1200 °C–4 h	Long straight fiber	Luminescent	[48]
Mullite	Al(C_3_H_7_O)_3_, Al(NO_3_)_3_·9H_2_O, Si(OC_2_H_5_)_4_	Sol-Gel	1000–1400 °C–2 h	Uniform-with beads	Reinforcement in ceramic matrix	[17]
Mullite	C_9_H_21_O_3_Al, (Al(NO_3_) 9H_2_O, SiC_8_H_20_O_4_	PVB	800–1400 °C–2 h	Straight	High temperature application,	[49]
Mn_2_O_3_ and Mn_3_O_4_	Manganese nitrate 4-hydrat	PVA	500, 700 and 1000 °C–1 h	Straight 3D porous random	Catalysis, ion exchange, molecular adsorption, biosensors, wastewater treatment and supercapacitors	[50]
Nb_2_O_5_	Metallic niobium powder	PVP	600–700 °C	Non-woven mat	Photocatalysis applications	[51]
NiO	Ni(NO_3_)_2_	PVP	400, 500 °C–1 h	Sintered particles, or lamellar after sintering	Gas sensor, Catalyst	[52]
NiO	Nickel (II) acetate tetrahydrate	SAN	500–700 °C–2 h	Straight	Thermistor	[53]
Ni/Al_2_O_3_	Ni(NO_3_)_2_·6H_2_O, Al(NO_3_)_3_·9H_2_O	PVP	700 to 1000 °C	Straight and smooth after calcination	Catalyst	[54]
Pr_0.4_Sr_0.6_Co_0.2_Fe_0.7_Nb_0.1_O_3−δ_	Pr(NO_3_)_3_·6H_2_O, Sr(NO_3_)_2_, Fe(NO_3_)_3_. 9H_2_O, Co(NO_3_)_3_·6H_2_O, H_3_[NbO(C_2_O_4_)_3_]	PVP	700 to 1000 °C–2 h	Short fibers	Solid oxide fuel cells	[55]
SiO_2_	Accuglass	PVP	400 °C several times	Bead shape fibers after heat treatment	Surface planarization	[56]
Silicon oxycarbide (SiOC)	Silicone resins (MK and H44 resin)	PVP	1000 °C–2 h	Straight and smooth	Mechanical application	[57]
Silicon oxycarbide (SiOC) doped Ag	Silver oxide or silver acetate, MK (polymethyl-silsesquioxane preceramic polymer)	PVP	1000 °C–2 h	Straight, Ag inside the fibers	Antibacterial activity, Gas permeability	[58]
SiO_2_ doped Bi_2_MoO_6_	(NH_4_)_6_Mo_7_O_24_·4H_2_O, Bi(NO_3_)_3_·5H_2_O	PVP	500–750 °C–2 h	Broken short fibers	Photocatalytic	[59]
SnO_2_	Tin acetate	PVAc	450 °C, 0.5 h	Regular fibrillar structure	Gas sensing	[60]
SnO_2_ doped Al	SnCl_2_·2H_2_O, Al(NO_3_)_3_·9H_2_O	PVP	600 °C–5 h	Bead shape fibers sintered after heat treatment	Hydrogen sensor	[61]
SnO_2_ doped Ce	SnCl_2_·2H_2_O, Ce(NO_3_)_3_·6H_2_O	PVP	600 °C–5 h	Hollow fibers	Ethanol gas sensor	[62]
SnO_2_ doped Eu	SnCl_2_·2H_2_O, Eu(NO_3_)_3_·6H_2_O	PVP	600 °C–5 h	Straight and smooth after calcination	Acetone sensor	[63]
Sm_2_O_3_	Samarium carbonate	PVA	1000 °C–2 h	Sintered particles forming a fiber	Optical film, insulator	[64]
SrFe_12_O_19_	Sr(NO_3_)_2_, Fe(NO_3_)_3_·9H_2_O	PVP	750 °C–1.5 h	Short and relatively dense after heat treatment	Photocatalytic adsorption	[65]
TiO_2_	Butyl titanate	PVP	550 °C–2 h	Smooth	Photocatalyst	[66]
TiO_2_	Titanium (IV) n-butoxide (TNBT)	PVP	500 °C–6 h	Depend on humidity varied from short to long fibers	Photocatalyst	[67]
TiO_2_	Ti(OiPr)_4_	Sol-Gel	500 °C–3 h	Short fibers	Electrochemical detection	[68]
WO_3_	WCl_6_, PVP		300–500 °C–1 h	Short fiber	NO_2_ gas responses	[69]
WO_3_	(NH_4_)6[H_2_W_12_O_40_] nH_2_O	PVP	500, 550, 600 °C–1 h	Short fiber with sintered NPs	N.A.	[70]
Yb_2_O_3_	Ytterbium chloride	CA	550 °C to 850 °C–2 h	Particle and agglomerate before and after calcination	fiber amplifiers, fiber optic technologies and lasers	[71]
ZnO	Zinc acetate dehydrate	PVA	500 and 700 °C–4 h	Straight, Fluffy surface	Biosensors	[72]
ZnO	Zinc acetate dehydrate	PVA	500 °C–2 h	Straight, Random	Low frequency AC electric fields	[21]
ZnO	Zinc nitrate hexahydrate	PVP	500 °C–3 h	Straight	Explosive nitro-compounds sensor	[73]
ZnO/BaO	Zinc acetate dehydrate barium acetate extra pure	PVA	850 °C–8 h	Straight	Electrical and non-linear optical	[74]
ZnO/SnO_2_	Zn(NO_3_)_2_·6H_2_O, SnCl_4_·5H_2_O	PAN	700–900 °C–3 h	Rough surface	Lithium-ion anode	[75]
ZnO doped Mg	Zinc acetate, magnesium acetate	PVA	300–600 °C–3 h	Sintered particles	Semiconductor	[76]
ZnO doped Cu	Zinc acetate, copper acetate	PVP	450 °C–3 h	Straight	Thermal and electrical conductivity, and optical properties	[77]
ZrC	Polyzirconoxane (PZO)	PAN	1400 °C–2 h	Core–shell, homogeneous	Ultra high temperature ceramics	[78]
ZrO_2_	Zirconium n-propoxide	PVA	600 to 1050 °C–4 h	Non-woven fibers	Thermal barrier coatings	[79]
ZrO_2_ (YSZ)	Zirconium oxychloride, Yttrium trinitrate hexahydrate	PVP	500–1500 °C	Bead shape fibers after heat treatment	Catalytic activity	[80]
ZrO_2_ (8YSZ)	ZrOCl_2_·8H_2_O, Y_2_O_3_	PVP	600–1400 °C–12 h	Hollow fibers	Catalytic combustion	[81]

**Table 2:** Recent products of electrspun composite ceramic/polymer NFs and their applications.

Ceramic	Polymer Type	Application	Ref
Graphene (G)	PANI, PS, DMF	Electrochemical sensor	[84]
TiO_2_	PVP	Photo catalyst	[85]
Al_2_O_3_	PVDF-CTFE	Lithium-ion batteries	[86]
Al_2_O_3_	PLA	Biomedical Application	[87]
ZrO_2_/Y_2_O_3_	PAN	Shielding in electronic device	[88]
HAp	PHBV	Tissue engineering	[89]
CNT	PVDF	Strain sensors	[90]
SiO_2_, Al_2_O_3_ or BaTiO_3_	P(VdF-HFP)	Lithium-ion batteries	[91]
BaTiO_3_	PVDF	Piezoelectric materials Energy harvesting	[92,93]
Boehmite (AlOOH)	PA6, PCL	Removal of heavy metal ions	[94]
CuO	PU	Electrical application	[95]
Sepiolite (Si_12_O_30_Mg_8_(OH)_4_–(H_2_O)_4.8_H_2_O)	PVB	Mechanical integrity in real applications	[96]

## Abbreviation

BMSC	Bone marrow mesenchymal stem cells	PDR	Parallel rotary disk
BSA	Bovine serum albumin	PE	Polyethylene
BJH	Barrett-Joyner-Halenda	PEDOT	Poly(3,4-ethylenedioxythiophene)
CA	Cellulose acetate	PEO	Polyethylene oxide
CF	Chloroform	PES	Polyethersulfone
CNF	Carbon Nano Fiber	PET	Polyethylene terephthalate
CNT	Carbon Nano Tube	PHBV	Poly(3-hydroxybutyrate-co-3-hydroxyvalerate)
CTFE	Chlorotrifluoroethylene	PLA	Poly(l-lactic) acid
DMF	Dimethylformamide	PLEDs	Polymer light-emitting diodes
DSC	Differential scanning calorimetry	PLGA	Poly(Lactide-co-Glycolide)
EMI	Electromagnetic interface	PMMA	Polymethylmethacrylate
FESEM	Field emission scanning electron microscopy	PPy	Polypyrrole
HA	Hydroxyapatite	PS	Polystyrene
IDEs	Interdigitated electrodes	PU	Polyurethane
I-DOPA	Levodopa	PVA	Polyvinylalcohol
ITO	Indium tin oxide	PVAc	Polyvinylacetate
LEDs	light-emitting diodes	PVB	Polyvinyl butyral
LIB	Lithium ion battery	PVC	Polyvinyl chloride
MB	Methylene blue	PVDF	Polyvinylidene fluoride
MIP	Mercury intrusion porosimetry	PVP	Polyvinylpyrrolidone
MO	Methylene orange	PZT	Lead zirconate titanate
MWCNT	Multi wall carbon nano tube	RhB	Rhodamine B
MX	Meloxicam	SAN	Poly(styrene-co-acrylonitrile)
NBs	Nano belts	SE	Shield effect
NCs	Nano crystallites	SEM	Scanning electron Microscopy
NFs	Nano fibers	SF	Solid fraction
NPLs	Nano plates	SOFC	Solid oxide fuel cell
NPs	Nano particles	SPEEK	Sulfonated polyether ether ketone
NTs	Nano tubes	STA	Simultaneous Thermal Analysis
NWs	Nano wires	TEM	Transition electron microscopy
P(VDF-HFD)	Poly(vinylidene fluoride-co-hexafluoropropylene)	TEOS	Tetraethoxysilane
PA6	Nylon 6	TG	Thermogravimetry
PA66	Nylon 66	TMWCNT	Treated multi wall carbon nano tube
PAN	Polyacrylonitrile	WGS	Water gas shift
PANI	Polyaniline	X-CT	X-ray computed tomography
PC	Polycarbonate	ZT	Thermoelectric properties
PCL	Polycaprolactone		

## References

[1] Zhou W., Long L., Xiao P., Li Y., Luo H., Hu W., Rui Y. (2017). Silicon carbide nano-fibers in-situ grown on carbon fibers for enhanced microwave absorption properties. Ceram. Int..

[2] Moradipour P., Dabirian F., Rajabi L., Ashraf Derakhshan A. (2016). Fabrication and characterization of new bulky layer mixed metal oxide ceramic nanofibers through two nozzle electrospinning method. Ceram. Int..

[3] Ramaseshan R., Sundarrajan S., Jose R., Ramakrishna S. (2007). Nanostructured ceramics by electrospinning. J. Appl. Phys..

[4] Brown T.D., Dalton P.D., Hutmacher D.W. (2016). Melt electrospinning today: An opportune time for an emerging polymer process. Prog. Polym. Sci..

[5] Hui W., Wei P., Dan L., He L. (2012). Electrospinning of ceramic nanofibers: Fabrication, assembly and applications. J. Adv. Ceram..

[6] Ikegame M., Tajima K., Aida T. (2003). Template Synthesis of Polypyrrole Nanofibers Insulated within One-Dimensional Silicate Channels: Hexagonal versus Lamellar for Recombination of Polarons into Bipolarons. Chem. Int. Ed..

[7] Hong Y., Legge R.L., Zhang S., Chen P. (2003). Effect of Amino Acid Sequence and pH on Nanofiber Formation of Self-Assembling Peptides EAK16-II and EAK16-IV. Biomacromolecules.

[8] Ma P.X., Zhang R. (1999). Synthetic nano-scale fibrous extracellular matrix. J. Biomed. Mater. Res..

[9] Ellison C.J., Phatak A., Giles D.W., Macosko C.W., Bates F.S. (2007). Melt blown nanofibers: Fiber diameter distributions and onset of fiber breakup. Polymer.

[10] Ondarçuhu T., Joachim C. (1998). Drawing a single nanofibre over hundreds of microns. EPL.

[11] Ramakrishna S., Ramalingam M., Sampath Kumar T.S. (2010). Biomaterials: A Nano Approach.

[12] Hong Q., Su W., Zhan H. (2013). Coaxial electrospun nanostructures and their applications. J. Mater. Chem. A.

[13] Sigmund W., Yuh J., Park H., Maneeratana V., Pyrgiotakis G., Daga A., Taylor J., Nino J.C. (2006). Processing and Structure Relationships in Electrospinning of Ceramic Fiber Systems. J. Am. Ceram. Soc..

[14] Biswas A., Park H., Sigmund W.M. (2012). Flexible ceramic nanofibermat electrospun from TiO_2_–SiO_2_ aqueous sol. Ceram. Int..

[15] Musiari F., Pirondi A., Morini F., Giuliese G., Zucchelli A., Brugo T.M., Minak G., Ragazzini C. (2016). Feasibility study of adhesive bonding reinforcement by electrospun nanofibers. Procedia Struct. Integr..

[16] Esfahani H., Prabhakaran M.P., Salahi E., Tayebifard A., Rahimipour M.R., Keyanpour-Rad M., Ramakrishna S. (2016). Electrospun nylon 6/zinc doped hydroxyapatite membrane for protein separation: Mechanism of fouling and blocking model. Mater. Sci. Eng. C.

[17] Chen Z., Zhao Z., Chen-Chih T., Konstantin K., Igor L., Fang M., Peng F. (2015). Electrospun mullite fibers from the sol–gel precursor. J. Sol-Gel Sci. Technol..

[18] Zhang H., Hang Y., Qin Y., Yang J., Wang B. (2014). Synthesis and characterization of sol–gel derived continuous spinning alumina based fibers with silica nano-powders. J. Eur. Ceram. Soc..

[19] Yaipimai W., Pornprasertsuk R. (2013). Fabrication of Pt, Pt–Cu, and Pt–Sn nanofibers for direct ethanol protonic ceramic fuel cell application. J. Mater. Sci..

[20] Ashraf A., Awad K., Asmari A. (2012). Wet-electrospun CuNP/carbon nanofibril composites: Potential application for micro surface-mounted components. Appl. Nanosci..

[21] Ghashghaie S., Bazargan A.M., Esmaeilpour Ganji M., Marzbanrad E., Zamani C., Raissi B., Keyanpour-rad M., Bahrevar M.A. (2011). An investigation on the behavior of electrospun ZnO nanofibers under the application of low frequency AC electric fields. J. Mater. Sci. Mater. Electron..

[22] Kim J.H., Yoo S.J., Kwak D.H., Jung H.J., Kim T.Y., Park K.H., Lee J.W. (2014). Characterization and application of electrospun alumina nanofibers. Nanoscale Res. Lett..

[23] Milanovic P., Dimitrijevic M., Heinemann R., Rogan J., Stojanovic D., Kojovic A., Aleksic R. (2013). Preparation of low cost alumina nanofibers via electrospinning of aluminium chloride hydroxide/poly (vinyl alcohol) solution. Ceram. Int..

[24] Zhang P., Jiao X., Chen D. (2013). Fabrication of electrospun Al_2_O_3_ fibers with CaO–SiO_2_ additive. Mater. Lett..

[25] Liu G.F., Zhang Z.D., Dang F., Cheng C.B., Hou C., Liu S.D. (2016). Formation and characterization of magnetic barium ferrite hollow fibers with low coercivity via co-electrospun. J. Magn. Magn. Mater..

[26] Calleja A., Sort J., Ricart J., Granados X., Palmer X., Roxana Vlad V., Puig T., Obradors X. (2016). Composite films combining electrospun fiber network and epitaxial oxide by chemical solution deposition. J. Sol-Gel Sci. Technol..

[27] Wang W., Chi N., Li Y., Yan W., Li X., Shao C. (2013). Electrospinning of magnetical bismuth ferrite nanofibers with photocatalytic activity. Ceram. Int..

[28] Cinar E., Koc S., It S., Aytimur A., Uslu I., Akdemir A. (2014). Synthesis, Characterization, and Thermoelectric Properties of Electrospun Boron-Doped Barium-Stabilized Bismuth-Cobalt Oxide Nanoceramics. Metall. Mater. Trans. A.

[29] Wang J., Yang Y., Huang Z., Kang F. (2011). Coaxial carbon nanofibers/MnO_2_ nanocomposites as freestanding electrodes for high-performance electrochemical capacitors. Electrochim. Acta.

[30] Qin D., Liang G., Gu A. (2013). CaCu_3_Ti_4_O_12_ electrospun fibre: A new form of CaCu_3_Ti_4_O_12_ and its dielectric property. J. Alloys Compd..

[31] Mohammadi M., Alizadeh P., Clemens F.J. (2016). Effect of using different precursors on electrospinning of CaCu_3_Ti_4_O_12_. Ceram. Int..

[32] Shamshi Hassan M., Amna T., SeobKhil M.Y. (2014). Synthesis of High aspect ratio CdTiO3 nanofibers via electrospinning: Characterization and photocatalytic activity. Ceram. Int..

[33] Sonsupap S., Kidkhunthod P., Chanlek N., Pinitsoontorn S., Maensiri S. (2016). Fabrication, structure, and magnetic properties of electrospun Ce_0.9_6Fe_0.04_O_2_ nanofibers. Appl. Surf. Sci..

[34] AbiJaoude M., Polychronopoulou K., Hinder S.J., Katsiotis M.S., Baker M.A., Greish Y.E., Alhassan S.M. (2016). Synthesis and properties of 1D Sm-doped CeO_2_ composite nanofibers fabricated using a coupled electrospinning and sol–gel methodology. Ceram. Int..

[35] Ju Y., Park J., Jung H., Cho S., Lee W. (2008). Fabrication and characterization of cobalt ferrite (CoFe_2_O_4_) nanofibers by electrospinning. Mater. Sci. Eng. B.

[36] Chiu T., Tu C., Chen Y. (2015). Fabrication of electrospun CuCr_2_O_4_ fibers. Ceram. Int..

[37] Schutz P., Alves A.K., Bergmann C.P. (2014). Effect of the in-air heat treatment in the phase formation and morphology of electrospun Cu_2_ZnSnS_4_ fibers. Ceram. Int..

[38] Jiang X., Xu H., Wang Q., Jiang L., Li X., Xu Q., Shi Y., Zhang Q. (2013). Fabrication of GdBaCo_2_O_5+d_ cathode using electrospun composite nanofibers and its improved electrochemical performance. J. Alloys Compd..

[39] Lei D., Qu B., Lin H.T., Wang T. (2015). Facile approach to prepare porous GeO_2_/SnO_2_ nanofibers via a single spinneret electrospinning technique as anodes for Lithium-ion batteries. Ceram. Int..

[40] Franco P.Q., Joao C.F.C., Silva J.C., Borges J.P. (2012). Electrospun hydroxyapatite fibers from a simple sol-gel system. Mater. Lett..

[41] Gibbons W.T., Liu T.H., Gaskell K.J., Jackson G.S. (2014). Characterization of palladium/copper/ceria electrospun fibers for water-gas shift catalysis. Appl. Catal. B Environ..

[42] Shim J., Lopez K.J., Sun H.-J., Park G., An J.-C., Eom S., Shimpalee S., Weidner J.W. (2015). Preparation and characterization of electrospun LaCoO_3_ fibers for oxygen reduction and evolution in rechargeable Zn-air batteries. Appl. Electrochem..

[43] Hayat K., Niaz F., Ali S., Iqbal M., Ajmal M., Ali M., Iqbal Y. (2016). Thermoelectric performance and humidity sensing characteristics of La_2_CuO_4_ nanofibers. Sens. Actuator B.

[44] Xu D., Luo L., Ding Y., Jiang L., Zhang Y., Ouyang X., Liu B. (2014). A novel nonenzymatic fructose sensor based on electrospun LaMnO_3_ Fibers. J. Electron. Chem..

[45] Yensano R., Pinitsoontorn S., Amornkitbamrung V., Maensiri S. (2014). Fabrication and Magnetic Properties of Electrospun La_0.7_Sr_0.3_MnO_3_ Nanostructures. J. Supercond. Nov. Magn..

[46] Yuan K., Feng C., Gan X., Yu Z., Wang X., Zhu L., Zhang G., Xu D. (2016). Fabrication of La_2_Zr_2_O_7_ ceramic fibers via electrospinning method using different La_2_O_3_ precursors. Ceram. Int..

[47] Sorour M.H., Rafei A.M.E.L., Hani H. (2016). Synthesis and characterization of electrospun aluminum doped Li_1.6_Mn_1.6_O_4_ spinel. Ceram. Int..

[48] Lu Q., Liu Q., Wei Q., Liu G., Zhuang J. (2013). Preparation and characterization of Lu_2_SiO_5_:Ce^3+^ luminescent ceramic fibers via electrospinning. Ceram. Int..

[49] Mohammad Ali Zadeh M., Keyanpour-Rad M., Ebadzadeh T. (2013). Synthesis of mullite nanofibres by electrospinning of solutions containing different proportions of polyvinyl butyral. Ceram. Int..

[50] Rafei A.M.E.L. (2015). Optimization of the electrospinning parameters of Mn_2_O_3_ and Mn_3_O_4_ nanofibers. Ceram. Int..

[51] Leindecker G.C., Alves A.K., Bergmann C.P. (2014). Synthesis of niobium oxide fibers by electrospinning and characterization of their morphology and optical properties. Ceram. Int..

[52] Ristic M., Marcius M., Petrovic Z., Music S. (2014). Dependence of NiO microstructure on the electrospinning conditions. Ceram. Int..

[53] George G., Anandhan S. (2014). Electrospun nickel oxide nanofiber webs for thermistor applications. Int. J. Plast. Technol..

[54] Wang Z., Hu X., Dong D., Parkinson G., Li C. (2017). Effects of calcination temperature of electrospun fibrous Ni/Al_2_O_3_ catalysts on the dry reforming of methane. Fuel Proc. Technol..

[55] Liu X., Li M., Wang Z., Zhang C., Xiong Y. (2016). Electro-spinning Pr_0.4_Sr_0.6_Co_0.2_Fe_0.7_Nb_0.1_O_3-δ_ nanofibers infiltrated with Gd_0.2_Ce_0.8_O_1.9_ nanoparticles as cathode for intermediate temperature solid oxide fuel cell. Ceram. Int..

[56] Mauro A.D., Fragala M.E. (2015). Electrospun SiO_2_ ‘‘necklaces’’ on unglazed ceramic tiles: A planarizing strategy. Superlattices Microst..

[57] Guo A., Roso M., Modesti M., Maire E., Adrien J., Colombo P. (2015). Characterization of porosity, structure, and mechanical properties of electrospun SiOC fiber mats. J. Mater. Sci..

[58] Guo A., Roso M., Colombo P., Liu J., Modesti M. (2015). In situ carbon thermal reduction method for the production of electrospun metal/SiOC composite fibers. J. Mater. Sci..

[59] Zhao J., Liu Z., Lu Q. (2016). Electrospun 1D SiO_2_ doped Bi_2_MoO_6_ microbelts for highly efficient photocatalytic applications. Dyes Pigment..

[60] Kim I., Jeon E.K., Choi S.H., Choi D.K., Tuller H.L. (2010). Electrospun SnO_2_ nanofiber mats with thermo-compression step for gas sensing applications. Electroceram.

[61] Xu X., Sun J., Zhang H., Wang X., Dong B., Jiang T., Wang W., Li Z., Wang C. (2011). Effects of Al doping on SnO_2_ nanofibers in hydrogen sensor. Sens. Actuator B.

[62] Mohanapriya P., Segawa H., Watanabe K., Watanabe K., Samitsu S., Natarajan T.S., Victor Jaya N., Ohashi N. (2013). Enhanced ethanol-gas sensing performance of Ce-doped SnO_2_ hollow nanofibers prepared by electrospinning. Sens. Actuator B.

[63] Jiang Z., Zhao R., Sun B., Nie G., Ji H., Lei J., Wang C. (2016). Highly sensitive acetone sensor based on Eu-doped SnO_2_ electrospun nanofibers. Ceram. Int..

[64] Panda P.K. (2013). Preparation and characterization of Samaria nanofibers by electrospinning. Ceram. Int..

[65] Li C.J., Wang J.N., Li X.Y., Zhang L.L. (2011). Functionalization of electrospun magnetically separable TiO_2_-coated SrFe_12_O_19_ nanofibers: Strongly effective photocatalyst and magnetic separation. J. Mater. Sci..

[66] Zhao K., Teng L., Tang Y., Chen X. (2014). Branched titanium oxide/vanadium oxide composite nanofibers formed by electrospinning and dipping in vanadium sol. Ceram. Int..

[67] Tikekar N.M., Lannutti J.J. (2012). Effects of humidity on titania-based polyvinylpyrolidone (PVP) electrospun fibers. Ceram. Int..

[68] Arvand M., Ghodsi N. (2014). Electrospun TiO_2_ nanofiber/graphite oxide modified electrode for electrochemical detection of l-DOPA in human cerebrospinal fluid. Sens. Actuator B.

[69] Giancaterini L., Emamjomeh S.M., Marcellis A., Palange E., Resmini A., Anselmi-Tamburini U., Cantalini C. (2016). The influence of thermal and visible light activation modes on the NO_2_ response of WO_3_ nanofibers prepared by electrospinning. Sens. Actuator B.

[70] Szilagyi I.M., Santala E., Heikkila M., Kemell M., Nikitin T., Khriachtchev L., Rasanen M., Ritala M., Leskela M. (2011). Thermal study on electrospun polyvinylpyrrolidone/ammonium metatungstate nanofibers: Optimising the annealing conditions for obtaining WO_3_ nanofibers. J. Therm. Anal. Calorim..

[71] Henriques M.S., Ferreira A.C., Cruz A., Ferreira L.M., Branco J.B., Brazda P., Jurek K., Stora T., Goncalves A.P. (2015). Preparation of Yb_2_O_3_ submicron- and nano-materials via electrospinning. Ceram. Int..

[72] Stafiniak A., Boratynsk B., Korczyc A.B., Szyszka A., Krasowska M., Prazmowska J., Fronc K., Elbaum D., Paszkiewicz R., Tłaczała M. (2011). A novel electrospun ZnO nanofibers biosensor fabrication. Sens. Actuator B.

[73] Cao Y., Zou X., Wang X., Qian J., Bai N., Li G.D. (2016). Effective detection of trace amount of explosive nitro-compounds by ZnO nanofibers with hollow structure. Sens. Actuator B.

[74] Nixon Samuel Vijayakumar G., Rathnakumar M., Sureshkumar P. (2012). Electrical and non-linear optical studies on electrospun ZnO/BaO composite nanofibers. Front. Mater. Sci..

[75] Luo L., Xu W., Xia Z., Fei Y., Zhu J., Chen C., Lu Y., Wei Q., Qiao H., Zhang X. (2016). Electrospun ZnO–SnO_2_ composite nanofibers with enhanced electrochemical performance as lithium-ion anodes. Ceram. Int..

[76] Sebnem Cetin S., Uslu I., Aytimur A., Ozcelik S. (2012). Characterization of Mg doped ZnO nanocrystallites prepared via Electrospinning. Ceram. Int..

[77] Wu Y., Dong Z., Jenness N.J., Clark R.L. (2011). In-situ formation of Cu metal crystals within nanostructured ZnO electrospun fibers. Mater. Lett..

[78] Li F., Kang Z., Huang X., Zhang G.J. (2014). Fabrication of zirconium carbide nanofibers by electrospinning. Ceram. Int..

[79] Singh S., Singh S., Vijayakumar M., Bhanu Prasad V.V. (2014). Electrospun ZrO_2_ fibers obtained from polyvinyl alcohol/zirconium n-propoxide composite fibers processed through halide free sol–gel route using acetic acid as a stabilizer. Mater. Lett..

[80] Chou C.C., Huang C.F., Yeh T.H. (2013). Characterization and catalytic activity of La_0.6_Sr_0.4_Co_0.2_Fe_0.8_O_3-d_–yttria stabilized zirconia electrospun nano-fiber as a cathode catalyst. Ceram. Int..

[81] Li J.Y., Tan Y., Xu F.M., Sun Y., Cao X.Q., Zhang Y.F. (2008). Hollow fibers of yttria-stabilized zirconia (8YSZ) prepared by calcination of electrospun composite fibers. Mater. Lett..

[82] Vivekanandhan S., Schreiber M., Kumar Mohanty A., Misra M., Pandey J.K. (2014). Advanced Electrospun Nanofibers of Layered Silicate Nano Composites: A Review of Processing, Properties, and Applications. Handbook of Polymer Nanocomposites, Processing, Performance and Application.

[83] Balasubramanian P., Roether J.A., Schubert D.W., Beier J.P., Boccaccini A.R. (2015). Bi-layered porous constructs of PCL-coated 45S5 bioactive glass and electrospun collagen-PCL fibers. J. Porous Mater..

[84] Promphet N., Rattanarat P., Rangkupan R., Chailapakul O., Rodthongkum N. (2015). An electrochemical sensor based ongraphene/polyaniline/polystyrene nanoporous fibers modifiedelectrode for simultaneous determination of lead and cadmium. Sens. Actuator B.

[85] Albetran H., Dong Y., Low I.M. (2015). Characterization and optimization of electrospun TiO_2_/PVP nano fibers using Taguchi design of experiment method. J. Asian Ceram. Soc..

[86] Lee Y.S., Jeong Y.B., Kim D.W. (2010). Cycling performance of lithium-ion batteries assembled with a hybrid composite membrane prepared by an electrospinning method. J. Power Sources.

[87] Kurtycz P., Ciach T., Olszyna A., Kunicki A., Radziun E., Roslon M., Dudkiewicz-Wilczynska J., Nowak K., Anuszewska E. (2013). Electrospun Poly(l-lactic)Acid/Nanoalumina (PLA/Al_2_O_3_) Composite Fiber Mats with Potential Biomedical Application—Investigation of Cytotoxicity. Fiber Polym..

[88] Sun Im J., Gu Kim J., Bae T.S., Lee Y.S. (2011). Effect of heat treatment on ZrO_2_-embedded electrospun carbon fibers used for efficient electromagnetic interference shielding. J. Phys. Chem. Sol..

[89] Suslu A., Albayrak A.Z., Urkmez A.S., Bayir E., Cocen U. (2014). Effect of surfactant types on the biocompatibility of electrospun HAp/PHBV composite nanofibers. J. Mater. Sci. Mater. Med..

[90] Xu Yan J.Z., Li M.M., Yu G.F., Zhang H.D., Pisula W., He X.X., Duvail J., Long Y.Z. (2015). Electrospun Aligned Fibrous Arrays and Twisted Ropes: Fabrication, Mechanical and Electrical Properties, and Application in Strain Sensors. Nanoscale Res. Lett..

[91] Raghavan P., Zhao X., Manuel J., Chauhan G.S., Ahn J.H., Ryu H.S., Ahn H.J., Kim K.W., Nah C. (2010). Electrochemical performance of electrospun poly(vinylidene fluoride-co-exafluoropropylene)-based nanocomposite polymer electrolytes incorporating ceramic fillers and room temperature ionic liquid. Electrochim. Acta.

[92] Lee C., Wood D., Edmondson D., Yao D., Erickson A.E., Tsao C.T., Revia R.A., Kim H., Zhang M. (2016). Electrospun uniaxially-aligned composite nanofibers as highly-efficient piezoelectric material. Ceram. Int..

[93] Nunes-Pereira J., Sencadas V., Correia V., Rocha J.G., Lanceros-Mendez S. (2013). Energy harvesting performance of piezoelectric electrospun polymer fibers and polymer/ceramic composites. Sens. Actuator A.

[94] Hota G., Kumar B.R., Ng W.G., Ramakrishna S. (2008). Fabrication and characterization of a boehmite nanoparticle impregnated electrospun fiber membrane for removal of metal ions. J. Mater. Sci..

[95] Nirmala R., Jeon K.S., Lim B.H., Navamathavan R., Kim H.Y. (2013). Preparation and characterization of copper oxide particles incorporated polyurethane composite nanofibers by electrospinning. Ceram. Int..

[96] Ben Hassan S.A., Stojanovic D.B., Kojovic A., Astvan I.A., Janackovic D., Uskokovic P.S., Aleksic R. (2014). Preparation and characterization of poly(vinylbutyral) electrospun nanocomposite fibers reinforced with ultrasonically functionalized sepiolite. Ceram. Int..

[97] Mohammadi M., Alizadeh P., Clemens F.J. (2015). Synthesis of CaCu_3_Ti_4_O_12_ nanofibers by electrospinning. Ceram. Int..

[98] Sangkhaprom N., Supaphol P., Pavarajarn V. (2010). Fibrous zinc oxide prepared by combined electrospinning and solvothermal techniques. Ceram. Int..

[99] Khalil A., Kim J.J., Tuller H.L., Rutledge G.C., Hashaikeh R. (2016). Gas sensing behavior of electrospun nickel oxide nanofibers: Effect of morphology and microstructure. Sens. Actuator B.

[100] Saleemi A.S., Abdullah A., Shah S.M.H., Anis-ur-Rehman M. (2013). Electrospun Proficient Polymer Based Nano Fibers with Ceramic Particles. J. Supercond. Nov. Magn..

[101] Starbova K., Petrov D., Starbov N., Lovchinov V. (2012). Synthesis of supported fibrous nanoceramics via electrospinning. Ceram. Int..

[102] Lamastra F.R., Nanni F., Menchini F., Nunziante P., Grilli M.L. (2015). Transparent nanostructured electrodes: Electrospun NiO nanofibers/NiO films. Thin Solid Film.

[103] Laudenslager M.J., Sigmund W.M. (2013). A continuous process to align electrospun nanofibers into parallel and crossed arrays. J. Nanopart. Res..

[104] Teo W.E., Ramakrishna S. (2006). A review on electrospinning design and nanofibre assemblies. Nanotechnology.

[105] Shin H.U., Ramsier R.D., Chase G.G. (2016). Influence of calcination temperature on the surface area of submicron-sized Al_2_O_3_ electrospun fibers. Appl. Phys. A.

[106] Dai Y., Liu W., Formo E., Sun Y., Xia Y. (2011). Ceramic nanofibers fabricated by electrospinning and their applications in catalysis, environmental science, and energy technology. Polym. Adv. Technol..

[107] Min J.W., Yim C.J., BinIm W. (2014). Preparation and electrochemical characterization of flower-like Li_1.2_Ni_0.17_Co_0.17_Mn_0.5_O_2_ microstructure cathode by electrospinning. Ceram. Int..

[108] Eick B.M., Youngblood J.P. (2009). SiC nanofibers by pyrolysis of electrospun preceramic polymers. J. Mater. Sci..

[109] Khalil K.A., Sherif E.M., Nabawy A.M., Abdo H.S., Marzouk W.W., Alharbi H.F. (2016). Titanium Carbide Nanofibers-Reinforced Aluminum Compacts, a New Strategy to Enhance Mechanical Properties. Materials.

[110] Guo A., Roso M., Modesti M., Liu J., Colombo P. (2014). Hierarchically structured polymer-derived ceramic fibers by electro spinning and catalyst-assisted pyrolysis. J. Eur. Ceram. Soc..

[111] Petrovic Z., Ristic M., Marcius M., MileIvanda Durina V., Music S. (2016). Hydrothermal processing of electrospun fibers in the synthesis of 1D ZnO nanoparticles. Mater. Lett..

[112] Sun Y., Li J.Y., Tan Y., Zhang L. (2009). Fabrication of aluminum nitride (AlN) hollow fibers by carbothermal reduction and nitridation of electrospun precursor fibers. J. Alloys Compd..

[113] Li J.Y., Sun Y., Tan Y., Xu F.M., Shi X.L., Ren N. (2008). Zirconium nitride (ZrN) fibers prepared by carbothermal reduction and nitridation of electrospun PVP/zirconium oxychloride composite fibers. Chem. Eng. J..

[114] Qin D., Liang G., Gu A., Yuan L. (2013). Facilely preparing various new titania electrospun fibers with controllable nanostructures using a three-step method. Sol-Gel Sci. Technol..

[115] Huang Z., Chen Y., Zhou W., Nie H., Hu Y. (2009). Preparation of silica hollow fibers by surface-initiated atom transfer radical polymerization from electrospun fiber templates. Mater. Lett..

[116] Fan X.X., He X.L., Li J.P., Gao X.J., Jia J. (2016). Ethanol sensing properties of hierarchical SnO_2_ fibers fabricated with electrospun polyvinylpyrrolidone template. Vacuum.

[117] Wang S.H., Wan Y., Sun B., Liu L.Z., Xu W. (2014). Mechanical and electrical properties of electrospun PVDF/MWCNT ultrafine fibers using rotating collector. Nanoscale Res. Lett..

[118] Chronakis I.S. (2005). Novel nanocomposites and nanoceramics based on polymer nanofibers using electrospinning process—A review. J. Mater. Process. Technol..

[119] Xiang C., Frey M.W. (2016). Increasing Mechanical Properties of 2-d-Structured Electrospun Nylon 6 Non-Woven Fiber Mats. Materials.

[120] Neppalli R., Causin V., Benetti E.M., Ray S.S., Esposito A., Wanjale S., Birajdar M., Saiter J.M., Marigo A. (2014). Polystyrene/TiO_2_ composite electrospun fibers as fillers for poly(butylene succinate-co-adipate): Structure, morphology and properties. Eur. Polym. J..

[121] Nanni F., Lamastra F.R., Pisa F., Gusmano G. (2011). Synthesis and characterization of poly(e-caprolactone) reinforced with aligned hybrid electrospun PMMA/nano-Al_2_O_3_ fibre mats by film stacking. J. Mater. Sci..

[122] Yang W., Sousa A.M.M., Thomas-Gahring A., Fan X., Jin T., Li T., Tomasula P.M., Liu L.S. (2016). Electrospun Polymer Nanofibers Reinforced by Tannic Acid/Fe^+++^ Complexes^+^. Materials.

[123] Gopal R., Kaur S., Ma Z., Chan C., Ramakrishna S., Matsuura T. (2006). Electrospun nano fibrous filtration membrane. J. Membr. Sci..

[124] Mota C., Labardi M., Trombi L., Astolfi L., Acunto M., Puppi D., Gallone G., Chiellini F., Berrettini S., Bruschini L. (2017). Design, fabrication and characterization of composite piezoelectric ultrafine fibers for cochlear stimulation. Mater. Des..

[125] Das P.P., Roy A., Tathavadekar M., Devi P.S. (2017). Photovoltaic and photocatalytic performance of electrospun Zn_2_SnO_4_ hollow fibers. Appl. Catal. B Environ..

[126] Gongda W., Kai W., Tingxiang R. (2014). Improved analytic methods for coal surface area and pore size distribution determination using 77 K nitrogen adsorption experiment. Int. J. Min. Sci. Technol..

[127] Bae J., Baek I., Choi H. (2017). Efficacy of piezoelectric electrospun nanofiber membrane for water Treatment. Chem. Eng. J..

[128] Mary A.W. (2006). Physical Properties of Materials.

[129] Cho D., Chen S., Jeong Y., Joo Y.L. (2015). Surface Hydro-properties of Electrospun Fiber Mats. Fiber Polym..

[130] Esfahani H., Prabhakaran M.P., Salahi E., Tayebifard A., Keyanpour-Rad M., Rahimipour M.R., Ramakrishna S. (2015). Protein adsorption on electrospun zinc doped hydroxyapatite containing nylon 6 membrane: Kinetics and isotherm. J. Colloid Interface Sci..

[131] Cho D., Lee S.G., Frey M.W. (2012). Characterizing zeta potential of functional nanofibers in a microfluidic device. J. Colloid Interface Sci..

[132] Raghavan P., Zhao X., Kim J.K., Manuel J., Chauhan G.S., Ahn J.H., Nah C. (2008). Ionic conductivity and electrochemical properties of nanocomposite polymer electrolytes based on electrospun poly(vinylidene fluoride-co-hexafluoropropylene) with nano-sized ceramic fillers. Electrochim. Acta.

[133] ASTM International Voluntary Organization (2010). Standard Test Method for Measuring the Electromagnetic Shielding Effectiveness of Planar Materials.

[134] Choi W., Choi L.S., Lee J.K., Yoon K.R. (2015). Preparation of fluorescein-functionalized electrospun fibers coated with TiO_2_ and gold nanoparticles for visible-light-induced photocatalysis. Mater. Chem. Phys..

[135] Xu Z., Li X., Wang W., Shi J., Teng K., Qian X., Shan M., Li C., Yang C., Liu L. (2016). Microstructure and photocatalytic activity of electrospun carbon nanofibers decorated by TiO_2_ nanoparticles from hydrothermal reaction/blended spinning. Ceram. Int..

[136] Dong X., Yang P., Liu Y., Jia C., Wang D., Wang J., Chen L., Che Q. (2016). Morphology evolution of one-dimensional ZnO nanostructures towards enhanced photocatalysis performance. Ceram. Int..

[137] Pascariu P., Airinei A., Olaru N., Olaru L., Nica V. (2016). Photocatalytic degradation of Rhodamine B dye using ZnO–SnO_2_ electrospun ceramic nanofibers. Ceram. Int..

[138] Yu H., Li Y., Song Y., Wu Y., Chen B., Li P. (2016). Preparation and luminescent properties of Gd_2_O_3_: Eu^3+^ nanofibres made by electrospinning. Ceram. Int..

[139] Liu B., Yan X., Yan H., Yao Y., Cai Y., Wei J., Chen S., Xu X., Li L. (2017). Preparation and Characterization of Mo Doped in BiVO_4_ with Enhanced Photocatalytic Properties. Materials.

[140] Malwal D., Gopinath P. (2016). Fabrication and Applications of Ceramic nanofibers in Water Remediation: A review. Environ. Sci. Technol..

[141] Roque-Ruiz J.H., Cabrera-Ontiveros E.A., Torres-Perez J., Reyes-Lopez S.Y. (2016). Preparation of PCL/Clay and PVA/Clay Electrospun Fibers for Cadmium (Cd^+2^), Chromium (Cr^+3^), Copper (Cu^+2^) and Lead (Pb^+2^) Removal from Water. Water Air Soil Pollut..

[142] Kim H.J., Pant H.R., Kim J.H., Choi N.J., Kim C.S. (2014). Fabrication of multifunctional TiO_2_–fly ash/polyurethane nanocomposite membrane via electrospinning. Ceram. Int..

[143] Zhe J., Leonard D., Altangerel Amarjargal T., Chan H.P., Kyoung-Jin A., Shon H.K., Cheol S.K. (2015). Removal of oil from water using magnetic bicomponent composite nanofibers fabricated by electrospinning. Compos. Part B.

[144] Brijesh K.S., Pradip K.D., Chitin C., Silk F. (2015). Electrospun Nanofibrous Scaffolds: A Prospective Approach for Regenerative Medicine. Polym. Compos. Mater..

[145] Meidanis H., Baciu D.E., Papavassiliou G., Fardis M.P. (2014). Electrospun ceramic and ceramic-polymer composite nanofibers for bone tissue engineering applications. J. Optoelectron. Adv. Mater..

[146] Li L., Li G., Jiang J., Liu X., Luo L., Nan K. (2012). Electrospun fibrous scaffold of hydroxyapatite/poly (e-caprolactone) for bone regeneration. Mater. Sci. Mater. Med..

[147] Zhang M., Liu Y., Jia Y., Han H., Sun D. (2014). Preparation and Evaluation of Electrospun Zein/HA Fibers Based on Two Methods of Adding HA Nanoparticles. J. Bionic Eng..

[148] Su C.J., Tu M.G., Wei L.J., Hsu T.T., Kao C.T., Chen T.H., Huang T.H. (2017). Calcium Silicate/Chitosan-Coated Electrospun Poly (Lactic Acid) Fibers for Bone Tissue Engineering. Materials.

[149] Liu H., Peng H., Wu Y., Zhang C., Cai Y., Xu G., Li Q., Chen X., Ji J., Zhang Y. (2013). The promotion of bone regeneration by nanofibrous hydroxyapatite/chitosan scaffolds by effects on integrin-BMP/Smad signaling pathway in BMSCs. Biomaterials.

[150] Falde E.J., Yohe S.T., Colson Y.L., Grinstaff M.W. (2016). Superhydrophobic materials for biomedical applications. Biomaterials.

[151] Yar M., Farooq A., Shahzadi L., Khan A.S., Mahmood N., Rauf A., Chaudhry A.A., Rehman I. (2016). Novel meloxicam releasing electrospun polymer/ceramic reinforced biodegradable membranes for periodontal regeneration applications. Mater. Sci. Eng. C Mater. Biol. Appl..

[152] Boakye M.A.D., Rijal N.P., Adhikari U., Bhattarai N. (2015). Fabrication and Characterization of Electrospun PCL-MgO-Keratin-Based Composite Nanofibers for Biomedical Applications. Materials.

[153] Münchow E.A., Pankajakshan D., Albuquerque M.T.P., Kamocki K., Piva E., Gregory R.L., Bottino M.C. (2016). Synthesis and characterization of CaO-loaded electrospun matrices for bone tissue engineering. Clin. Oral. Investig..

[154] Padmavathi R., Sangeetha D. (2013). Synthesis and characterization of electrospun carbon nano fiber supported Pt catalyst for fuel cells. Electrochim. Acta.

[155] Pradipta S., Sudeshna B., Sunita M. (2015). Synthesis and Sensing characterization of ZnO nanofibers prepared by Electrospinning. Mater. Today Proc..

[156] Wei S., Zhao J., Du W. (2015). Synthesis, characterization and acetone-sensing properties of bristlegrass-like ZnO nanostructure. Ceram. Int..

[157] Petronela P., Anton A., Niculae O., Iulian P., Valentin N., Liviu S., Florin T. (2016). Microstructure, electrical and humidity sensor properties of electrospun NiO–SnO_2_ nanofibers. Sens. Actuators B.

[158] Yin M., Yang F., Wang Z., Zhu M., Liu M., Xu X., Li Z. (2017). A Fast Humidity Sensor Based on Li^+^-Doped SnO_2_ One-Dimensional Porous Nanofibers. Materials.

[159] Arafat M.M., Haseeb A.S.M.A., Akbar S.A., Quadir M.Z. (2017). In-situ fabricated gas sensors based on one dimensional core-shell TiO_2_-Al_2_O_3_ nanostructures. Sens. Actuators B.

[160] Yongjin J., Kyuhong L., Kinam K., Sunghwan K. (2016). Pore-Structure-Optimized CNT-Carbon Nanofibers from Starch for Rechargeable Lithium Batteries. Materials.

[161] Gao T., Le T., Yang Y., Yu Z., Huang Z., Kang F. (2017). Effects of Electrospun Carbon Nanofibers’ Interlayers on High-Performance Lithium–Sulfur Batteries. Materials.

[162] Kimura K., Matsumoto H., Hassoun J., Panero S., Scrosati B., Tominaga Y. (2015). A Quaternary Poly(ethylene carbonate)-Lithium Bis (trifluoromethanesulfonyl)imide-Ionic Liquid-Silica Fiber Composite Polymer Electrolyte for Lithium Batteries. Electrochim. Acta.

[163] Prasanth R., Jae-Won C., Jou-Hyeon A., Gouri C., Ghanshyam S., Chauhan H.-J.A., Changwoon N. (2008). Novel electrospun poly(vinylidene fluoride-co-hexafluoropropylene)—In situ SiO_2_ composite membrane-based polymer electrolyte for lithium batteries. J. Power Sources.

[164] Jeong H.S., Kim D.W., Jeong Y.U., Lee S.Y. (2010). Effect of phase inversion on microporous structure development of Al_2_O_3_/poly(vinylidene fluoride-hexafluoropropylene)-based ceramic composite separators for lithium-ion batteries. J. Power Sources.

[165] Wu N., Cao Q., Wang X., Li S., Li X., Deng H. (2011). In situ ceramic fillers of electrospun thermoplastic polyurethane/poly(vinylidene fluoride) based gel polymer electrolytes for Li-ion batteries. J. Power Sources.

[166] Li X., Chen Y., Huang H., Mai Y.W., Zhou L. (2016). Electrospun carbon-based nano structured electrodes for advanced energy storage, A review. Energy Storage Mater..

[167] Baji A., Mai Y.W., Li Q., Liu Y. (2011). Nanoscale investigation of ferroelectric properties in electrospun barium titanate/polyvinylidene fluoride composite fibers using piezoresponse force microscopy. Compos. Sci. Technol..

[168] Huang Y., Wang Y., Gao L., He X., Liu P., Liu C. (2017). Characterization of stretchable SWCNTs/Lycra fabric electrode with dyeing process. J. Mater. Sci. Mater. Electron..

[169] Gajendiran M., Choi J., Kim S.J., Kim K., Shin H., Koo H.J., Kyobum K. (2017). Conductive Biomaterials for Tissue Engineering Applications. J. Ind. Eng. Chem..

[170] Li C., Li Q., Ni X., Liu G., Cheng W., Han G. (2017). Coaxial Electrospinning and Characterization of Core-Shell Structured Cellulose Nanocrystal Reinforced PMMA/PAN Composite Fibers. Materials.

[171] Gaminian H., Montazer M. (2017). Decorating silver nanoparticles on electrospun cellulose nanofibers through a facile method by dopamine and ultraviolet irradiation. Cellulose.

[172] Liu L., Li S., Guo X., Wang L., Liu L., Wang X. (2016). The fabrication of In_2_O_3_ nanowire and nanotube by single nozzle electrospinning and their gas sensing property. J. Mater. Sci. Mater. Electron..

[173] Chiu T.W., Chen Y.T. (2015). Preparation of CuCrO_2_ nanowires by electrospinning. Ceram. Int..

[174] Swallow M., Luo J.K., Siores E., Patel I., Dodds D. (2008). A piezoelectric fibre composite based energy harvesting device for potential wearable applications. Smart Mater. Struct..

[175] Wu W., Bai S., Yuan M., Qin Y., Wang Z.L., Jing T. (2012). Lead Zirconate Titanate Nanowire Textile Nanogenerator for Wearable Energy-Harvesting and Self-Powered Devices. Am. Chem. Soc..

[176] Stoppa M., Chiolerio A. (2014). Wearable Electronics and Smart Textiles: A Critical Review. Sensors.

[177] Hu W.P., Zhang B., Zhang J., Luo W.L., Guo Y., Chen S.J., Yun M.J., Ramakrishna S., Long Y.Z. (2017). Ag/alginate nanofiber membrane for flexible electronic skin, accepted Manuscript. Nanotechnology.

[178] Lee K.S., Shim J., Park M., Kim H.Y., Son D.I. (2017). Transparent nanofiber textiles with intercalated ZnO@graphene QD LEDs for wearable electronics. Compos. Part B.

[179] Park M., Lee K.S., Shim J., Liu Y., Lee C., Cho H., Kim M.J., Park S.J., Yun Y.J., Kim H.Y. (2016). Environment friendly, transparent nanofiber textiles consolidated with high efficiency PLEDs for wearable electronics. Org. Electron..

[180] Dong L., Liang G., Xu C., Liu W., Pan Z.Z., Zhou E., Kang F., Yang Q.H. (2017). Multi Hierarchical Construction-induced Superior Capacitive Performances of Flexible Electrodes for Wearable Energy Storage, accepted Manuscript. Nano Energy.

[181] Li Z., Shen J., Abdalla I., Yu J., Ding B. (2017). Nanofibrous Membrane Constructed Wearable Triboelectric Nanogenerator for High Performance Biomechanical Energy Harvesting. Nano Energy.

[182] Yao Y., Li J., Lu H., Gou J., Hui D. (2015). Investigation into hybrid configuration in electrospun nafion/silica Nanofiber. Compos. Part B.

[183] Gao B., Zuo L., Zuo B. (2016). Sound Absorption Properties of Spiral Vane Electrospun PVA/nano Particle Nanofiber Membrane and Non-woven Composite Material. Fiber Polym..

